# Bidirectional cingulate-dependent danger information transfer across rats

**DOI:** 10.1371/journal.pbio.3000524

**Published:** 2019-12-05

**Authors:** Yingying Han, Rune Bruls, Efe Soyman, Rajat Mani Thomas, Vasiliki Pentaraki, Naomi Jelinek, Mirjam Heinemans, Iege Bassez, Sam Verschooren, Illanah Pruis, Thijs Van Lierde, Nathaly Carrillo, Valeria Gazzola, Maria Carrillo, Christian Keysers

**Affiliations:** 1 Netherlands Institute for Neuroscience, Royal Netherlands Academy of Arts and Sciences, Amsterdam, the Netherlands; 2 A student of Vrije Universiteit Amsterdam, Amsterdam, the Netherlands; 3 A student of the Department of Applied Life Sciences, FH Campus Wien, Wien, Austria; 4 Department of Psychology, Faculty of Social and Behavioural Sciences, University of Amsterdam (UvA), Amsterdam, the Netherlands; 5 A student of the Faculty of Psychology and Educational Sciences, Ghent University, Ghent, Belgium; Emory University, UNITED STATES

## Abstract

Social transmission of freezing behavior has been conceived of as a one-way phenomenon in which an observer “catches” the fear of another. Here, we use a paradigm in which an observer rat witnesses another rat receiving electroshocks. Bayesian model comparison and Granger causality show that rats exchange information about danger in both directions: how the observer reacts to the demonstrator’s distress also influences how the demonstrator responds to the danger. This was true to a similar extent across highly familiar and entirely unfamiliar rats but is stronger in animals preexposed to shocks. Injecting muscimol in the anterior cingulate of observers reduced freezing in the observers and in the demonstrators receiving the shocks. Using simulations, we support the notion that the coupling of freezing across rats could be selected for to more efficiently detect dangers in a group, in a way similar to cross-species eavesdropping.

## Abstract 2

This study shows that when one rat witnesses another receive electric shocks, the rats transfer information in both directions in a way that is independent of familiarity but is increased by prior experience with shocks. Simulations show that a phenomenon typically associated with empathy and prosociality may actually have evolved to serve the more selfish purpose of danger detection.

## Introduction

**Fig 1:**
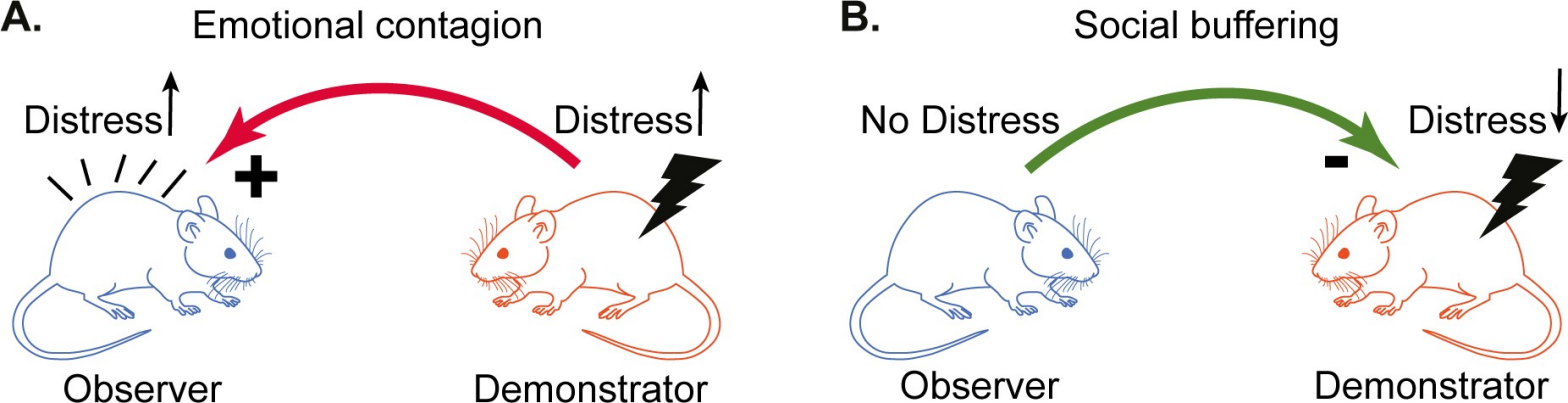
Emotional contagion and social buffering paradigms. (A) A schematic representation of typical paradigms used to investigate emotional contagion. An observer rat witnesses a demonstrator rat receive an electric foot shock. The shock induces fear and pain responses in the demonstrator, which in turn is unidirectionally transferred to the observer, which shows increased freezing thought to indicate an increase in distress. In these paradigms, the variable of interest is the amount of freezing of the observer. (B) A schematic representation of the social buffering paradigm. A demonstrator rat receives an electric foot shock. The fear response of the demonstrator, freezing in particular, is reduced in the presence of an observer rat. The variable of interest is the amount of freezing of the demonstrator.

**Fig 2:**
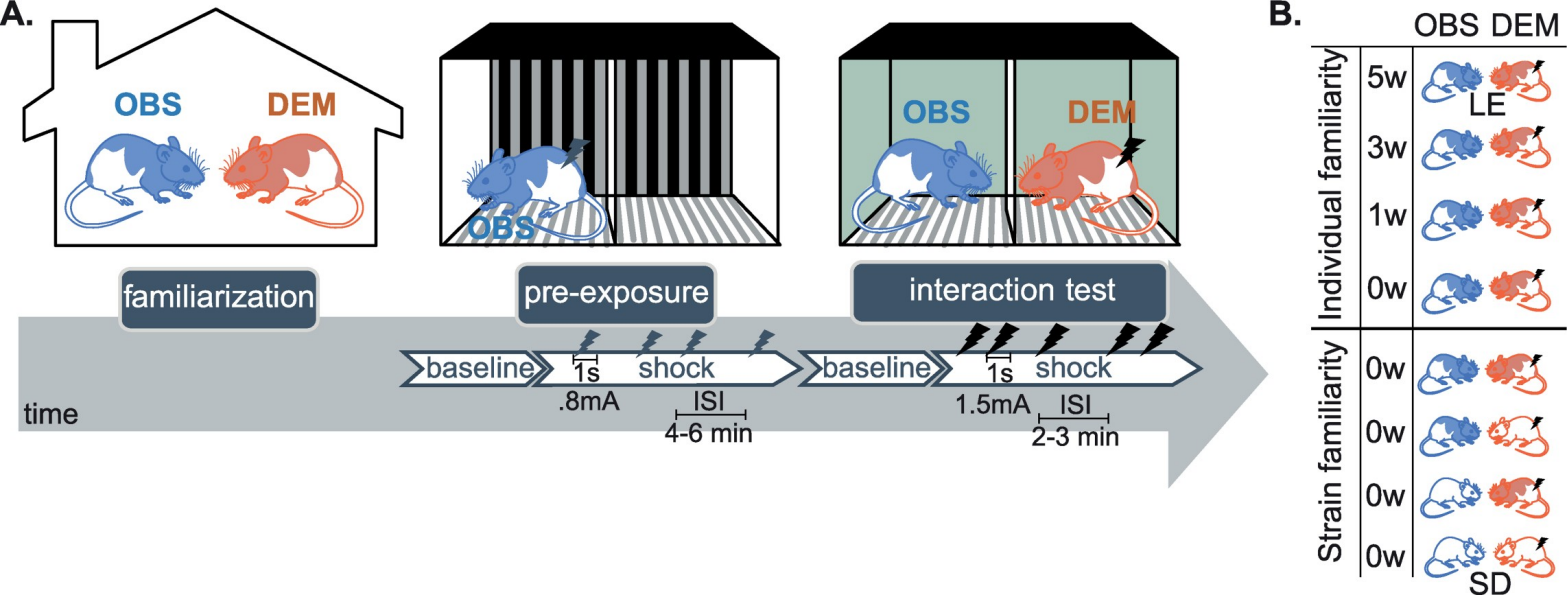
Experimental procedure. The procedure started with a familiarization phase (left) in which a DEM (in orange) was housed together with an OBS (in blue) for different periods of time depending on the experimental group (rightmost table). After the familiarization phase, the OBSs were preexposed to foot shocks alone (middle). The preexposure procedure consisted of a 12-minute baseline and a 12-minute shock period in which the observer received four foot shocks (0.8 mA, 1 second each, ISI: 4–6 minutes). This was followed by the interaction test (right), consisting of a 12-minute baseline and a 12-minute shock period. During the shock period, the observer witnessed the demonstrator in an adjacent chamber receive five foot shocks (1.5 mA, 1 second each, ISI: 2–3 minutes). In the individual familiarity experiment, all animals were LE (hooded rats in the right table) and varied in how familiar they were with the particular individual they observe in the interaction test. In the strain familiarity experiment, the observer–demonstrator dyads were either from the same strain (i.e., both hooded LE or both albino SD) or from different strains (i.e., one hooded LE and one albino SD), and thus varied in their familiarity with the strain they observe. DEM, demonstrator rat; ISI, intershock interval; LE, Long-Evans; OBS, observer rat; SD, Sprague Dawley.

The ability to anticipate threats and deploy defensive responses appropriately is key to survival [1]. Following extensive evidence that humans witnessing the pain of others show (1) brain activity as if they had been in pain themselves [2–5] and (2) defensive changes in cortical excitability reminiscent of the freezing behavior found in animals [6,7], two separate streams of research have shown that rodents are also sensitive to the emotional state of others (Fig 1). First, rats and mice freeze not only after they experience an unescapable shock themselves but also when witnessing another animal receive such a shock [8–14]. Second, rodents freeze less to a fear-conditioned stimulus when they are paired with a conspecific that does not engage in freezing [15–25]. These observations suggest that rodents have evolved mechanisms to more selectively deploy defensive behavior in anticipation of a danger by using the response of conspecifics as evidence for or against the presence of the danger. Here, we introduce a combination of an established behavioral paradigm and advanced analysis methods to systematically quantify the transfer of information across rats in the context of danger (Fig 2). We first use this combination to study whether familiarity across animals and prior exposure to foot shocks influence the magnitude of this information transfer. Next, we use this methodology to quantify the impact of deactivating the anterior cingulate cortex (ACC) on this information transfer.

The paradigm we developed in the laboratory involves a shock-experienced observer rat interacting through a perforated transparent divider with a demonstrator rat receiving foot shocks. We quantify the freezing behavior of both animals during an initial 12-minute baseline period and a 12-minute test period in which the demonstrator receives five foot shocks (1.5 mA, 1 second each, intershock interval [ISI]: 240–360 seconds, Fig 2). We then introduce Bayesian model fitting, model comparison, and Granger causality to quantify the degree to which the freezing of the demonstrator influences the freezing of the observer and, vice versa, whether the freezing of the observer influences the freezing of the demonstrator. Unlike more traditional analysis methods that focus on one direction of information flow at a time (Fig 1), the methods we introduce allow us to capture social influences in both directions in the same paradigm and thereby address the need to study true social interactions [26]. We then test whether information indeed flows in both directions and quantify the effect of familiarity, prior exposure to foot shocks, and anterior cingulate deactivation.

We explore the effect of familiarity because this paradigm was initially developed to study affective empathy and emotional contagion. The term emotional contagion can be traced back to the German *Stimmungsuebertragung*, introduced by Konrad Lorentz to refer to cases in which witnessing a conspecific in a particular emotion, expressed via movements and sounds, triggers a similar emotion in the witness (“der Anblick des Artgenossen in bestimmten Stimmungen, die sich durch Ausdrucksbewegungen und -laute äußern können, im Vogel selbst eine ähnliche Stimmung hervorruft” [27]). The term empathy is used more variably. In the human literature, it is defined as feeling what another feels while “being aware of the causal mechanism that induced the emotional state in that other” [28] or, put otherwise, “when we know that the other person's affective state is the source of our own affective state” [2]. In the rodent literature, some argue empathy can be used more generally as “the capacity to be affected by and share the emotional state of another” irrespective of the ability to know who is the source of the emotion [29]. Many believe empathy evolved in the context of parental care, in which feeling the distress of offspring motivates nurturing and thereby increases Darwinian fitness [28,30,31]. As such, empathy could be expected to be stronger toward individuals that are closer or more familiar to us, although this familiarity bias is not part of the definition of empathy. The effect of familiarity on emotional contagion has thus been studied in mice [10,11,32,33]. These mouse studies show that increasing the level of familiarity across demonstrator and observer mice increases how much the demonstrator influences the observer. It should be noted that in mice, encountering an unfamiliar conspecific triggers a stress response via glucocorticoids, and this stress response appears to reduce emotional contagion [33]. Does familiarity also influence information transfer in rats? In contrast to mice, no studies have tested the role of familiarity in the behavioral response of rats directly witnessing a conspecific experience a painful stimulus. What we do know is that interactions with a conspecific that had been exposed to a painful stimulus elsewhere can lead to stronger effects in more-familiar individuals [34] or in siblings [35]. However, because the imminence of a threat changes the behavioral and neural responses of an animal [36], transmission of a state influenced by past danger signals (potentially via olfactory cues) differs from witnessing an acute reaction to distress (partially via auditory and visual cues). Finally, rats will help trapped individuals from a strain they are familiar with more than animals from a strain they are unfamiliar with [37], but such prosocial behavior may be more tightly regulated because of its potential cost than emotional contagion.

We explore the effect of prior exposure to foot shocks because our own experiments have shown that, for female rats, prior exposure was necessary to trigger a strong freezing response when rats observe shocks delivered to a demonstrator [8]. Also, Greene has observed that helping is stronger in preexposed rats [38]. In contrast, although some work has been done in preexposed mice [39], most of the emotional contagion work in mice has been done with shock-naïve animals made to observe a demonstrator receive 20 shocks [11,12]. We therefore performed a small experiment to quantify whether information transfer is significant even across rats that have not received foot shocks and how the magnitude of that transfer compares with that of preexposed animals.

We also deactivate the ACC (area 24 in particular), a region that contains emotional mirror neurons in rodents [40,41], to test its impact on our measures of information transfer. Finally, we perform simulations to explore how information transfer across individuals can aid the detection of danger from a signal detection perspective.

## Results

### General behavioral responses

**Fig 3:**
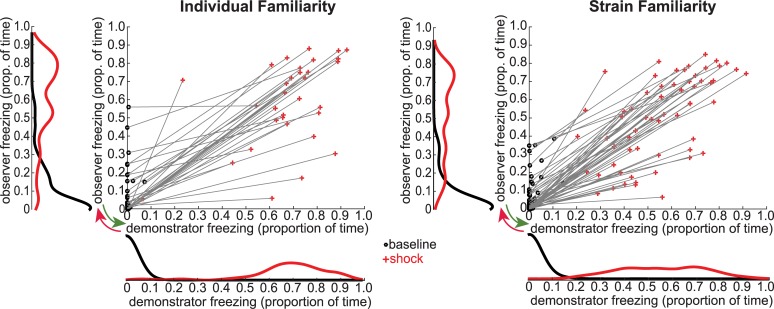
Observer and demonstrator freezing. For both the individual familiarity and the strain familiarity experiments, the scatter plots indicate the prop. of time spent freezing by the demonstrator (x-axis) and observer (y-axis) during both baseline (black dots) and the shock period (red pluses). The marginal histograms indicate the distribution of freezing behavior during baseline (black lines) and the shock period (red lines) using a kernel with 0.05 bandwidth. Red arrow: possible influence of the demonstrator freezing on the observer freezing (akin to emotional contagion). Green arrow: possible influence of the observer freezing on the demonstrator freezing (akin to social buffering if the level of the observer freezing is lower than that of the demonstrator). Data can be found at http://dx.doi.org/10.17632/h8fkyr2z35.1. prop., proportion.

We manipulated familiarity in two ways. In the first experiment (individual familiarity experiment), all demonstrator–observer dyads were from the same strain (i.e., Long-Evans) but differed in how long they had been housed together with that particular individual. In the second experiment (strain familiarity experiment), all demonstrator–observer dyads were unfamiliar with the animal they were paired with during the interaction test but differed in whether rats were familiar with the strain of their pair-mate (i.e., both Long-Evans or both Sprague Dawley) or were unfamiliar with that strain (i.e., one Long-Evans and one Sprague Dawley). The scatter plots of Fig 3 show, for both the individual familiarity and strain familiarity experiments, how much observers and demonstrators froze during the 12-minute baseline (when no shock was delivered, black dots) and during the 12-minute shock period, during which the demonstrator received 5 shocks (red plusses). In both experiments, the freezing during baseline (black dots) is higher among the observers than the demonstrators (two-tailed independent-samples *t* test, baseline freezing observers versus demonstrators, individual familiarity experiment, t_(62)_ = 3.6, *p* < 0.001, strain familiarity experiment, t_(118)_ = 3.9, *p* < 0.001). Preexposing the observers to shocks in a different environment thus led to some generalization of fear to the test environment. This effect was, however, modest, with average observer baseline freezing below 10% in both experiments (individual familiarity experiment, mean = 9.4% ± standard error of the mean [SEM] = 2.5%, strain familiarity experiment, mean = 6.4% ± SEM = 1.3%). The elongated shape of the scatter plots during the shock period (red plusses) suggests a relationship between the freezing levels of demonstrators and observers: the dyads in which the demonstrators froze the most are often the dyads in which the observers also froze the most. To explore the directionality of this relationship, we use Bayesian modeling and Granger causality.

### Effect of familiarity and feedback—Bayesian model comparison

**Fig 4:**
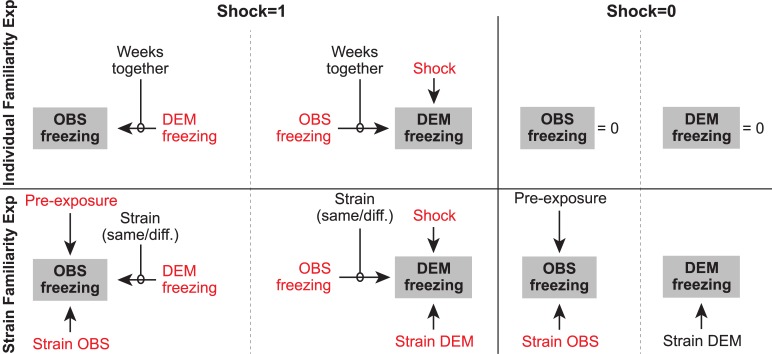
Variables included in the Bayesian models. Several models were built, separately for the individual and strain familiarity experiments, based on the factors that could describe the observer and demonstrator freezing. Here, the full models for the individual familiarity experiment (top) and strain familiarity experiment (bottom) are shown separately for epochs in which shocks are delivered (shock = 1) and those in which no shocks are delivered (shock = 0). The target variables that the models explain are shown in a gray box. These full models were then compared against simplified models, and the variables included in the winning model are shown in red. The modulator “Weeks together” captures whether the effect across animals depends on the number of weeks the observer and demonstrator spent together before testing (i.e., 0, 1, 3, 5 weeks). This modulator was implemented in two different ways (see Table 1A and 1B, and Fig 5): (1) in a way that models a linear increase of interindividual influence with number of weeks spent together, with the impact thus five times stronger after 5 compared with 1 week spent together, or (2) in a way that simply models a different connection weight for each group. Strain OBS and strain DEM capture the effect of a particular strain on the average freezing level of that strain. Strain (same/diff.) is a binary variable indicating whether the observer and demonstrator dyad were of the same or different strain. Finally, the variable preexposure indicates the amount of freezing of the observer during preexposure. Unfortunately, we only collected movies during preexposure in the strain familiarity experiment and thus cannot retrospectively include that variable in the models of the individual familiarity experiment. Data can be found at http://dx.doi.org/10.17632/h8fkyr2z35.1. DEM, demonstrator rat; diff., different; Exp, experiment; OBS, observer rat.

Bayesian modeling was used to (1) compare models with feedback, in which the freezing of the observer influences how much the demonstrator freezes, against models without feedback and (2) identify whether familiarity influences the coupling between the animals. Separate models were constructed using different combinations of experimental variables that could explain the observer’s and demonstrator’s freezing in the two experiments. Fig 4 summarizes the variables included in the models, with those that significantly explain the observer’s and demonstrator’s freezing marked in red.

#### Results from the individual familiarity experiment

Of the eight tested models designed to explain demonstrator freezing, the best one (model 6, expected log pointwise predictive density according to the leave-one-out approximation [Elpd_loo_] estimate = 41.5, standard error [SE] = 15.2; Table 1A) shows that Freezing_dem_ = 0.47 × Shock_dem_ + 0.37 × Freezing_obs_ × Shock_dem_. This indicates that, within our paradigm, the freezing of the demonstrator (Freezing_dem_) is approximated by assuming that it is 0 when no shock is delivered (since the variable Shock_dem_ is then equal to 0, nulling all elements of the equation). However, when a shock is delivered, the demonstrator’s freezing can be estimated at 0.47 (i.e., the demonstrator freezes 47% of the time) if the observer does not freeze at all plus 0.37 times the freezing of the observer if the observer does freeze. That the freezing of the observer was part of the model best explaining the data suggests that—unlike what a classic one-way perspective would assume—information is indeed exchanged in both directions, with the behavior of the demonstrator influenced by that of the observer and that of the observer influenced by that of the demonstrator. Indeed, the feedback parameters in the models all have 95% credibility intervals not encompassing 0, which provides additional evidence that even the somewhat weaker influence from the observer back to the demonstrator was significant.

**Table 1:** Model comparisons for the individual and strain familiarity experiments.

**A. Explaining Demonstrator Freezing in the Individual Familiarity Experiment**												
**Model**	**1**	**4**	**3**	**2**	**5**	**8**	**7**	**6**				
**Elpd**_**loo**_ **Estimate**	−27.2	−23.0	16.4	20.5	36.0	36.1	37.1	**41.5**				
**SE**	1.9	6.7	10.3	11.2	16.4	14.7	13.5	**15.2**				
**Intercept**_**obs**_	0.34 (0.26–0.42)	---	---	---	---	---	---	**---**				
**Shock**_**dem**_	---	---	---	---	0.68 (0.64–0.73)	0.64 (0.57–0.71)	0.47 (0.35–0.58)	**0.47** **(0.36–0.58)**				
**Freezing**_obs_*Shock_**dem**_	---	---	---	1.05 (0.96–1.15)	---	---	---	**0.37** **(0.19–0.54)**				
Weeks*Freezing_obs_*Shock_**dem**_	---	0.31 (0.24–0.38)	---	---	---	0.03 (0.0–0.07)	---	**---**				
0Weeks*Freezing_obs_*Shock_**dem**_	---	---	0.94 (0.74–1.13)	---	---	---	0.29 (0.08–0.49)	**---**				
1Week*Freezing_obs_*Shock_**dem**_	---	---	1.14 (0.94–1.34)	---	---	---	0.42 (0.19–0.64)	**---**				
3Weeks*Freezing_obs_*Shock_**dem**_	---	---	1.04 (0.87–1.20)	---	---	---	0.39 (0.20–0.59)	**---**				
5Weeks*Freezing_obs_*Shock_**dem**_	---	---	1.13 (0.88–1.37)	---	---	---	0.34 (0.09–0.60)	**---**				
**Sigma**_**dem**_	0.37 (0.31–0.44)	0.34 (0.29–0.41)	0.18 (0.15–0.21)	0.17 (0.14–0.20)	0.13 (0.11–0.16)	0.13 (0.11–0.15)	0.12 (0.10–0.14)	**0.12** **(0.10–0.14)**				
**B. Explaining Observer Freezing in the Individual Familiarity Experiment**												
**Model**	**4**	**1**	**3**	**2**								
**Elpd**_**loo**_ **Estimate**	−21.8	−17.9	10.7	**13.5**								
**SE**	7.0	3.0	7.9	**7.8**								
**Intercept**_**dem**_	---	0.34 (0.25–0.44)	---	**---**								
**Freezing**_dem_*Shock_**dem**_	---	---	---	**0.85** **(0.75–0.94)**								
Weeks*Freezing_dem_*Shock_**dem**_	0.24 (0.18–0.30)	---	---	**---**								
0Weeks*Freezing_dem_*Shock_**dem**_	---	---	0.87 (0.66–1.08)	**---**								
1Week*Freezing_dem_*Shock_**dem**_	---	---	0.77 (0.59–0.94)	**---**								
3Weeks*Freezing_dem_*Shock_**dem**_	---	---	0.93 (0.76–1.10)	**---**								
5Weeks*Freezing_dem_*Shock_**dem**_	---	---	0.77 (0.54–1.00)	**---**								
**Sigma**_**obs**_	0.34 (0.28–0.40)	0.32 (0.27–0.38)	0.20 (0.16–0.24)	**0.19** **(0.16–0.23)**								
**C. Explaining Demonstrator Freezing in the Strain Familiarity Experiment**												
**Model**	**1**	**7**	**2**	**3**	**9**	**8**	**4**	**10**	**5**	**6**	**11**	**12**
**Elpd**_**loo**_ **Estimate**	−26.7	−13.3	65.4	66.2	70.5	71.6	78.0	87.9	103.8	108.2	**108.8**	110.0
**SE**	4.4	10.4	12.5	12.2	11.4	11.5	9.8	12.6	12.4	12.2	**13.0**	13.1
**Intercept**_**dem**_	0.28 (0.23–0.34)	---	---	---	---	---	---	---	---	---	**---**	---
**Shock**_**dem**_	---	---	---	---	---	---	0.55 (0.52–0.59)	0.48 (0.44–0.52)	0.32 (0.26–0.38)	0.33 (0.27–0.39)	**0.30** **(0.25–0.36)**	0.31 (0.25–0.37)
**Strain**_dem_*Shock_**dem**_	---	0.63 (0.53–0.73)	---	---	0.13 (0.06–0.20)	0.14 (0.07–0.21)	---	0.15 (0.09–0.21)	---	---	**0.10** **(0.05–0.15)**	0.08 (0.02–0.13)
**Strain**_dem_*NoShock_**dem**_	---	0.02 (−0.08 to 0.1)	---	---	0.00 (−0.05 to 0.06)	0.01 (−0.04 to 0.06)	---	0.02 (−0.02 to 0.06)	---	---	**0.01** **(−0.03 to 0.05)**	0.00 (−0.04 to 0.04)
**Freezing**_obs_*Shock_**dem**_	---	---	0.99 (0.93–1.06)	---	---	0.86 (0.78–0.95)	---	---	0.46 (0.35–0.57)	---	**0.40** **(0.29–0.51)**	---
SameStrain*Freezing_obs_*Shock_**dem**_	---	---	---	1.06 (0.97–1.14)	0.90 (0.78–1.02)	---	---	---	---	0.53 (0.41–0.64)	**---**	0.46 (0.34–0.58)
DifferentStrain*Freezing_obs_*Shock_**dem**_	---	---	---	0.92 (0.83–1.01)	0.84 (0.74–0.94)	---	---	---	---	0.37 (0.25–0.49)	**---**	0.35 (0.24–0.47)
**Sigma**_**dem**_	0.30 (0.27–0.34)	0.27 (0.24–0.31)	0.14 (0.12–0.16)	0.14 (0.12–0.13)	0.13 (0.12–0.15)	0.13 (0.12–0.15)	0.13 (0.11–0.14)	0.11 (0.10–0.13)	0.10 (0.09–0.11)	0.10 (0.08–0.11)	**0.09** **(0.08–0.11)**	0.09 (0.08–0.11)
**D. Explaining Observer Freezing in the Strain Familiarity Experiment**												
**Model**	**4**	**1**	**7**	**10**	**3**	**2**	**9**	**8**	**6**	**5**	**12**	**11**
**Elpd**_**loo**_ **Estimate**	−60.7	−19.7	−1.1	−0.8	56.1	56.8	65.2	65.4	66.9	67.6	71.0	**71.2**
**SE**	7.3	5.1	11.3	11.2	9.5	9.8	9.0	9.5	9.0	9.2	8.5	**8.8**
**Intercept**_**obs**_	---	0.28 (0.23–0.33)	---	---	---	---	---	---	---	---	---	**---**
**Strain**_obs_*Shock_**dem**_	---	---	0.58 (0.50–0.66)	0.61 (0.53–0.69)	---	---	0.13 (0.06–0.20)	0.13 (0.06–0.20)	---	---	0.05 (−0.04 to 0.13)	**0.04** **(−0.04 to 0.13)**
**Strain**_obs_*NoShock_**dem**_	---	---	0.09 (0.01–0.17)	0.10 (0.02–0.19)	---	---	0.08 (0.03–0.13)	0.08 (0.03–0.13)	---	---	0.09 (0.04–0.13)	**0.08** **(0.04–0.13)**
**Preexposure**_obs_*Shock_**dem**_	0.10 (0.0–0.21)	---	---	−0.07 −0.13 to 0.00	---	---	---	---	0.09 (0.05–0.12)	0.09 (0.05–0.12)	0.08 (0.04–0.12)	**0.08** **(0.04–0.12)**
**Preexposure**_obs_*NoShock_**dem**_	0.01 (−0.09 to 0.11)	---	---	−0.02 (−0.08 to 0.05)	---	---	---	---	0.01 (−0.02 to 0.05)	0.01 (−0.02 to 0.05)	−0.01 (−0.05 to 0.03)	**−0.01** **(−0.05 to 0.03)**
**Freezing**_DEM_*Shock_**dem**_	---	---	---	---	---	0.90 (0.83–0.96)	---	0.76 (0.66–0.86)	---	0.89 (0.83–0.95)	---	**0.85** **(0.74–0.95)**
SameStrain*Freezing_dem_*Shock_**dem**_	---	---	---	---	0.85 (0.77–0.94)	---	0.71 (0.60–0.82)	---	0.85 (0.77–0.93)	---	0.80 (0.69–0.91)	**---**
DifferentStrain*Freezing_dem_*Shock_**dem**_	---	---	---	---	0.96 (0.86–1.07)	---	0.83 (0.71–0.95)	---	0.96 (0.86–1.05)	---	0.91 (0.79–1.03)	**---**
**Sigma**_**obs**_	0.40 (0.35–0.46)	0.30 (0.27–0.34)	0.24 (0.21–0.28)	0.24 (0.21–0.27)	0.15 (0.13–0.17)	0.15 (0.13–0.17)	0.14 (0.12–0.16)	0.13 (0.12–0.15)	0.14 (0.12–0.16)	0.14 (0.12–0.16)	0.13 (0.11–0.15)	**0.13** **(0.11–0.15)**

For each experiment, separate models were constructed to describe the level of freezing of the dem and the obs. The number of models varies depending on the variables that were included (Fig 4). The models were ordered based on their increasing Elpd_loo_, with the worst model on the left and the best model on the right. The first column lists the variables included in each model. Values in the table indicate the parameter estimates with their credible intervals below (2.5%–97.5%). The model in bold always indicates the winning model.

Abbreviations: dem, demonstrator; Elpd_loo_, expected log pointwise predictive density according to the leave-one-out approximation; obs, observer; SE, standard error of the Elpd_loo_

**Fig 5:**
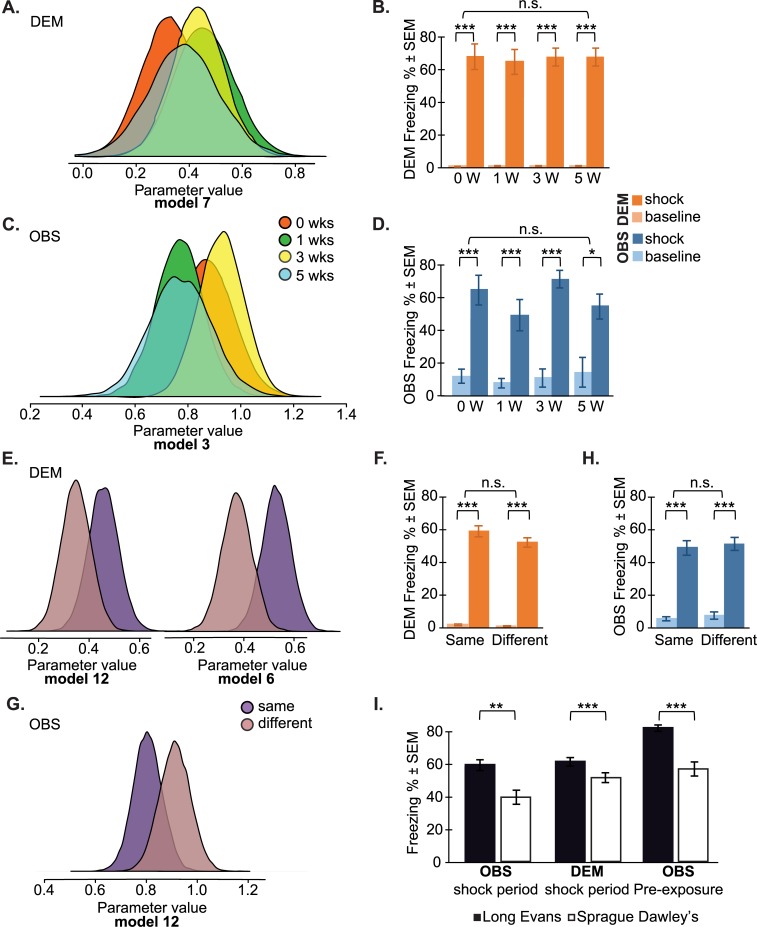
Parameter estimates and model-free analysis. (A) Parameter estimates of the influence from OBS → DEM from model 7 in Table 1A as a function of weeks spent together. Note the considerable overlap and shift away from 0, illustrating the lack of a familiarity effect and the consistent feedback from the observer, respectively. (B) Model-free comparison across the familiarity groups. (C, D) Same as panels A and B, but using observer freezing as the dependent variable. (E, F, G, H) Same for the strain familiarity experiment. (I) Long-Evans rats froze more than Sprague Dawleys both in a social context during the shock period of the strain familiarity experiment and when tested alone during shock preexposure. For all pairwise comparisons, *t* test, *p < 0.05, **p < 0.01, ***p < 0.001. n.s. refers to the absence of a significant group × epoch interaction in an ANOVA (see text for details). Data can be found at http://dx.doi.org/10.17632/h8fkyr2z35.1. DEM, demonstrator rat; OBS, observer rat.

As expected, delivery of foot shocks is a key variable that induces freezing in the demonstrator. In contrast, none of the familiarity variables were present in the model with the best fit, indicating that familiarity does not modulate the freezing of demonstrators sufficiently to improve the predictive performance of the model. This was true independent of whether familiarity was modeled to vary linearly with weeks (model 8, in which more weeks spent together would increase the influence of the other animal’s freezing) or nonlinearly (model 7, in which a different strength of influence from the observer freezing is calculated for each familiarity level). Indeed, inspecting the distribution of the parameters for the social feedback fitted separately for each group in model 7 (distributions in Fig 5A) shows substantial overlap between the credibility intervals for these parameters. Put differently, the data do not provide evidence that the freezing of an unfamiliar observer (0_weeks_) has a significantly smaller effect than that of more-familiar observers. In addition, even for the 0_weeks_ group, the social feedback parameter has a distribution that is shifted away from 0, suggesting significant social feedback onto even unfamiliar demonstrators. Note that, in all models, the observer’s freezing was only considered as a predictor, whereas the demonstrator received shocks (i.e., Freezing_obs_*Shock_dem_), and models that considered the freezing of the observer without the presence of a shock (e.g., during baseline) performed less well.

The findings from these model comparisons were confirmed with traditional group-level analysis: the inclusion of Shock_dem_ as a significant parameter is reflected in a significant increase of freezing during shock compared with baseline for each group (Fig 5B), and the lack of familiarity effect is compatible with the outcome of a 2 epoch (baseline versus shock) × 4 familiarity (0, 1, 3, 5 weeks) ANOVA that revealed a main effect of epoch (F_[1,28]_ = 409.685, *p* < 0.0001) but a lack of significant main effect of familiarity (F_[3,28]_ = 0.569, *p* = 0.64) or familiarity × epoch interaction (F_[3,28]_ = 0.463, *p* = 0.711).

Of the four defined models explaining the observers’ freezing, the best one estimated that Freezing_obs_ = 0.85 × Freezing_dem_ × Shock_dem_ (model 2, Elpd_loo_ estimate = 13.5, SE = 7.8, Table 1B). This shows that, within a dyad, the freezing of the observer (Freezing_obs_) is coupled to that of the demonstrator (Freezing_dem_) with a high gain of 0.85 × Freezing_dem_. In other words, the freezing of the observer is only 15% lower than that of the demonstrator receiving the actual shocks. Inspecting the distribution of the parameters influenced by familiarity of model 3 reveals high overlap between the distributions, with all of them having credibility intervals not encompassing 0 (Fig 5C). This further reinforces the notion that a strong linkage exists independently of the familiarity level. Traditional group-level comparisons (Fig 5D) confirm that administering a shock to the demonstrator has a strong effect on the observer but that familiarity does not modulate this effect: a 2 epoch (baseline versus shock) × 4 familiarity (0, 1, 3, 5 weeks) ANOVA showed a main effect of epoch (F_[1,28]_ = 113.069, *p* < 0.0001) but no main effect of familiarity (F_[3,28]_ = 1.214, *p* = 0.323) or epoch × familiarity interaction (F_[3,28]_ = 1.135, *p* = 0.352).

Together, the Bayesian model comparisons on the individual familiarity experiment data therefore show (1) that embracing a bidirectional model of information transmission improves our ability to explain the data and (2) that there is no apparent change in the intensity of the bidirectional coupling as a function of how long Long-Evans rats were pair-caged. The mutual influence evidenced here occurs for unfamiliar and familiar animals alike.

#### Results from the strain familiarity experiment

Unlike the individual familiarity experiment, in which all animals were Long-Evans rats, to further test the impact of familiarity, the strain familiarity experiment included rats of different strains: Long-Evans and Sprague Dawleys. The difference in strain could impact freezing in two ways. Much like in the first experiment, strain influences how familiar the partners are with the strain of their counterpart: Long-Evans rats were highly familiar with Long-Evans rats but had never been in contact with Sprague Dawley rats. In addition, one of the two strains may in general freeze more in response to a stressor (be it social or nonsocial) than the other. This second consideration motivated us to include a number of factors in addition to those included in the first experiment (see Fig 4). In addition to (1) the freezing percent of observers and demonstrators and (2) whether or not the demonstrators received foot shocks (baseline versus shock period), the following predictors were also included: (3) a binary variable capturing the strain (Long-Evans or Sprague Dawley) of the observers as a predictor for observer freezing (Strain_obs_), (4) a binary variable capturing the strain (Long-Evans or Sprague Dawley) of the demonstrator to predict demonstrator freezing (Strain_dem_), and (5) a binary variable that captured cases in which the two rats were of the same strain (SameStrain) and those in which they were of different strains (DifferentStrain). Finally, to capture individual differences in freezing behavior, which is crucial for predicting observer freezing, we also analyzed freezing during the initial preexposure of the observer rats, in which they experienced a number of shocks alone, and used that as a predictor of how much they would respond to seeing another rat receive shocks (Preexposure_obs_, Fig 3).

Comparing the fit of the models tested to explain the demonstrators’ freezing (Table 1C) reveals a group of four models with Elpd_loo_ > 100 (models 5, 6, 11, 12). Compared with those with much poorer fits, these models all incorporate whether the demonstrator received a shock (Shock_dem_) and feedback from how strongly the observer froze (Freezing_obs_, either modulated by strain difference or not). Because the difference between the model fits across these four models is small compared with the SE of the model fit, we explored the parameter estimates to determine whether there was evidence for an effect of strain. To determine whether there was an effect of strain on the effect of the shock, we examined the credibility intervals of the Strain_dem_*Shock_dem_ parameter in models 11 and 12. In both cases, the credibility intervals did not include 0, suggesting a small but significant effect of strain on the amount of freezing following the shock, with the Long-Evans reacting to the shock with higher freezing than the Sprague Dawleys. To determine whether there was an effect of same/different strain on the feedback from the observer, we compared the credibility intervals for the SameStrain*Freezing_obs_*Shock_dem_ versus the DifferentStrain*Freezing_obs_*Shock_dem_ parameters from model 12 (Fig 5E, Table 1C). The posterior distributions overlap so much, and the difference in the model fit between model 11 and 12 is so small, that this does not provide robust evidence for a strain effect. We thus consider model 11 as the best description of our data: Freezing_dem_ = 0.30 × Shock_dem_ + 0.10 × Strain_dem_ × Shock_dem_ + 0.40 × Freezing_obs_ × Shock_dem_ (model 11 Elpd_loo_ estimate = 108.8, SE = 13.0, Table 1C). This means that if no shock is being delivered, the estimated freezing is 0, because of the “× Shock_dem_” behind all terms. If a shock is delivered, Freezing_dem_ is then estimated at 0.30 plus 0.10 if the demonstrator is a Long-Evans plus 0.40 × the freezing of the observer when dyads are from same or different strains. The observer’s freezing was only a good predictor when the demonstrator actually received shocks (i.e., Freezing_obs_*Shock_dem_), and models that considered the freezing of the observer without the presence of a shock (e.g., during baseline) performed less well.

The difference in freezing between strains during the shock period is confirmed by group-level analyses showing that Long-Evans demonstrators froze significantly more compared with Sprague Dawley demonstrators (Fig 5I). Importantly, as for the individual familiarity experiment, the feedback parameters (i.e., those including Freezing_obs_) all had credibility intervals excluding 0, providing evidence for the presence of a sizable feedback effect. Additional model-free group-level analyses (Fig 5F) confirm these findings: there was a significant increase of freezing levels during shock compared with baseline and no effect of familiarity: a 2 epoch (baseline versus shock) × 4 familiarity (0, 1, 3, 5 weeks) ANOVA showed a main effect of epoch (F_[1,58]_ = 637.323, *p* < 0.0001) but no main effect of familiarity (F_[1,58]_ = 2.491, *p* = 0.12) or epoch × familiarity interaction (F_[1,58]_ = 1.695, *p* = 0.198).

**Fig 6:**
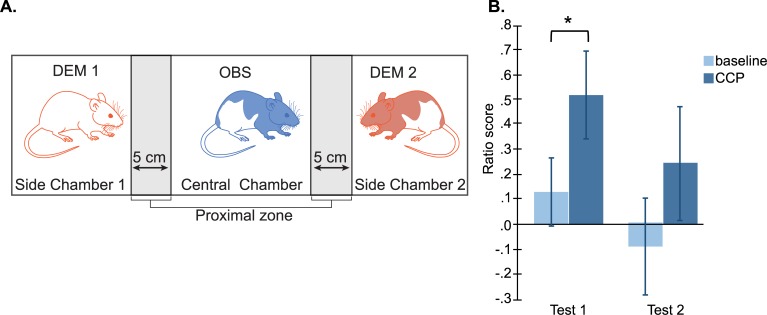
Same-strain recognition experiment. For this experiment, eight observers (OBS: all Long-Evans, four of which also served as demonstrators) and eight demonstrators (DEM; four Long-Evans and four Sprague Dawley rats) were used. (A) The test was conducted in a three-chamber testing box consisting of one large central chamber (L:72 cm × W:33 cm) and two small side chambers (each: L:27 cm × W:33 cm). The central chamber was separated from the side chambers by transparent perforated walls. The day prior to testing, all animals were habituated to the testing box. The test consisted of a 5-minute baseline period, in which observers were individually placed in the central compartment, followed by a 10-minute CPP, in which two unfamiliar demonstrators (DEM1 and DEM2: one Long-Evans and one Sprague Dawley rat) were simultaneously placed in one of the side compartments (placement was randomized). To avoid bias, the placement of the demonstrators occurred when the observer was in the center zone of the central compartment. Each observer had two tests, separated by an interval of 30 minutes to 1 hour, in which the location of the Sprague Dawley and Long-Evans rats was changed. The amount of time that the observers spent in the proximal zone during the initial 90 seconds of the baseline and CPP was scored, and a ratio score was estimated (difference in the time spent in the proximal zone of the Long-Evans rat and the time spent in the proximal zone of the Sprague Dawley rat divided by the sum of the time the observer spent in the proximal zone of the Long-Evans and Sprague Dawley rat. (B) Results show that, in test 1 and 2, observers spent more time in the proximal zone of the same-strain (Long-Evans) than of the different-strain (Sprague Dawley) demonstrator rats compared with baseline. This was significant for the first test of each trio (paired samples *t* test, test 1: t_(6)_ = 2.58, p_2tail_ = 0.04; test 2: t_(6)_ = 1.64, p_2tail_ = 0.15, *p < 0.05). Data can be found at http://dx.doi.org/10.17632/h8fkyr2z35.1. CCP, choice preference period; DEM, demonstrator rat; L, length; OBS, observer rat; W, width.

For the observers, the model best explaining the data was Freezing_obs_ = 0.08 × Strain_obs_ × NoShock_dem_ + 0.08 × Preexposure_obs_ × Shock_dem_ + 0.85 × Freezing_dem_ × Shock_dem_ (model 11, Elpd_loo_ estimate = 71.2, SE = 8.8, Table 1D), showing that within a dyad, the freezing of the observer (Freezing_obs_) during the shock period is strongly modulated by the freezing of the demonstrator (Freezing_dem_ × Shock_dem_) and more weakly by the preexposure of the observer (Preexposure_obs_ × Shock_dem_). During the no-shock period (i.e., baseline), the freezing of the observer is mildly modulated by the strain of the observer animal (Strain_obs_ × NoShock_dem_), which suggests possible differences between freezing levels of observers of different strains (Fig 5I). Whether observer–demonstrator dyads were from the same or different strains, however, did not modulate the strength of the coupling between the demonstrator’s and observer’s freezing. An additional experiment, which showed that Long-Evans observers are capable of distinguishing same (other unfamiliar Long-Evans rats) from different strains (unfamiliar Sprague Dawley rats) under dim red light conditions (i.e., same as in the strain familiarity experiment) confirmed that this lack of effect was not due to the possibility that the observers could not distinguish the two strains (Fig 6). The lack of modulation by the same/different strain is supported not only by the fact that splitting the effect of Freezing_dem_ of model 12 into the same/different strain does not outperform model 11 but also by the fact that the same- versus different-strain versions of the parameter estimates (Fig 5G) overlap considerably. This illustrates that the behavior of the observer is modulated by that of the demonstrator, regardless of whether they are from the same or different strain. This is further supported by an analysis showing no difference in freezing levels between same- and different-strain dyads (Fig 5H, left side): a 2 epoch (baseline versus shock) × 4 familiarity (0, 1, 3, 5 weeks) ANOVA showed a main effect of epoch (F_[1,58]_ = 269.113, *p* < 0.0001) but no main effect of familiarity (F_[1,58]_ = 0.284, *p* = 0.596) or epoch × familiarity interaction (F_[1,58]_ = 0.004, *p* = 0.953).

Despite differences in experimental manipulations, both experiments suggest that there is robust bidirectional information transfer within observer–demonstrator dyads: (1) the freezing level of an observer is better predicted when taking the freezing of the demonstrator into account, (2) the freezing level of a demonstrator is better predicted when taking the freezing of the observer into account, and (3) estimates of the coupling parameters have credibility intervals not including 0. In contrast, the familiarity level does not improve predictions significantly, and the coupling parameters for different familiarity levels (individual or strain) overlap. This was true when familiarity was manipulated at the individual level in terms of weeks spent together and at the strain level in terms of whether animals were familiar with the strain of their partner.

#### Moment-to-moment coupling—Granger causality

The results of Bayesian modeling provide evidence for bidirectional information transfer between observers and demonstrators. Dyads with higher overall observer freezing are dyads with higher demonstrator freezing despite receiving the same shock. If the freezing of the observer truly influences that of the demonstrator, as the Bayesian models suggest, we would expect to find evidence of such bidirectional influence at the level of the moment-to-moment fluctuations of freezing in individuals: fluctuations in the freezing of the demonstrator should be explained (in the statistical sense) by earlier fluctuations of the observer at a second-by-second timescale. Granger causality analyses were used to examine this prediction [42,43].

To have an overview of the information flow within the observer–demonstrator dyads during the interaction test, Granger causality was computed including all dyads from both the individual and strain familiarity experiments. Granger causality values (i.e., Granger F values) were calculated separately for the baseline and the shock period. During baseline, significant Granger causality was found in both directions: from the demonstrator to the observer (Granger F = 0.086, *p* < 0.0001) and from the observer to the demonstrator (Granger F = 0.156, *p* < 0.0001), meaning that there is time-coupled bidirectional information flow. The order of the Granger causality model is determined automatically by the analysis and was 21, suggesting that the freezing of an animal is influenced by the freezing levels in the past 21 seconds. In addition, the observer-to-demonstrator Granger causality was numerically larger than in the opposite direction, which can be explained by the influence of the preexposure on the observer's freezing: because the observers were preexposed to foot shocks, they showed some contextual fear generalization to the test setup and more spontaneous freezing during the baseline (Fig 3, black marginal histograms). This baseline freezing potentially influenced the demonstrators. Conversely, as the demonstrators froze less during baseline, they could not have as much influence on the observers. For the shock period, significant Granger causality was also found in both directions: from the demonstrator to the observer (Granger F = 0.059, *p* < 0.0001) and from the observer to the demonstrator (Granger F = 0.035, *p* < 0.0001). As expected, delivery of foot shocks to the demonstrator makes the information flow from the demonstrator to the observer stronger compared with the opposite direction, as indicated by the larger Granger causality values in the demonstrator-to-observer direction.

**Fig 7:**
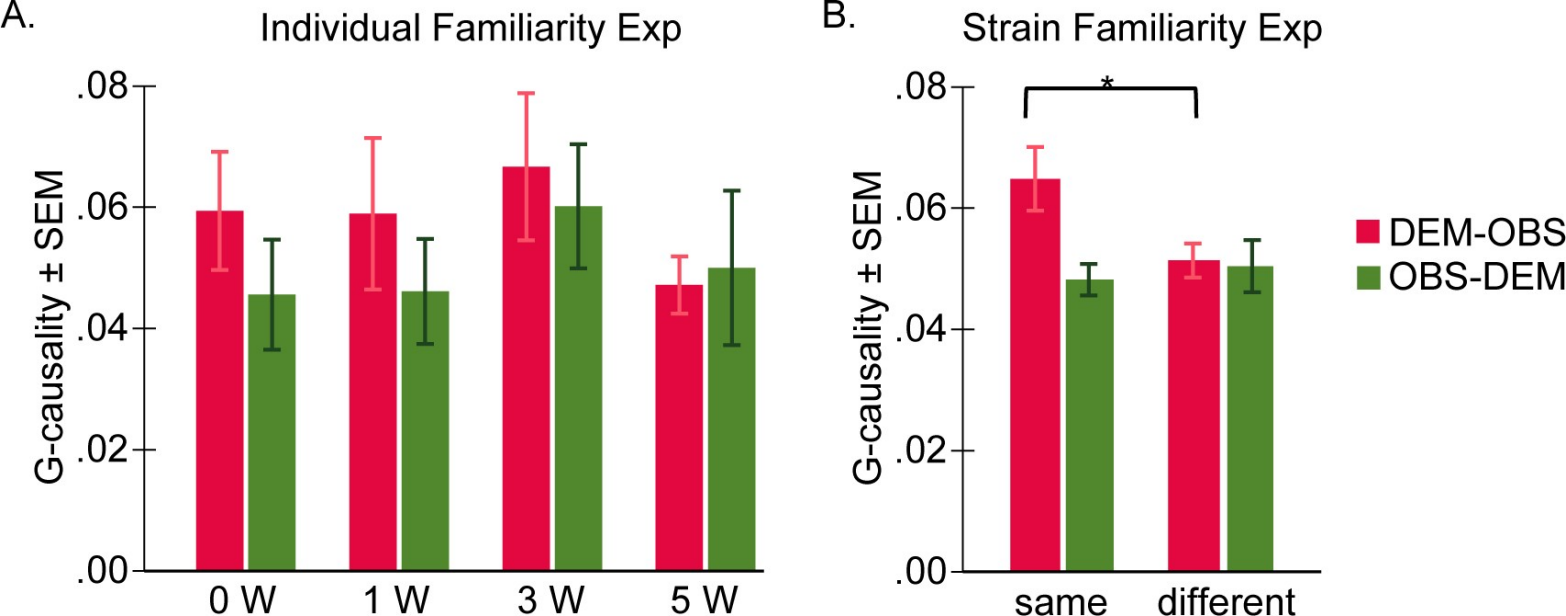
Directionality of information transfer. (A) G-causality results. Mean ± SEM for G-causality values for the demonstrator-to-observer direction (DEM–OBS, in red) and the observer-to-demonstrator direction (OBS–DEM, in green) during the shock period, for the individual familiarity experiment. (B) G-causality results for the strain familiarity experiment. Data can be found at http://dx.doi.org/10.17632/h8fkyr2z35.1. DEM, demonstrator rat; different, different strain; Exp, experiment; G-causality, Granger causality; OBS, observer rat; same, same strain; SEM, standard error of the mean; W, week.

To investigate the effect of familiarity, Granger causality between the demonstrator's and the observer's freezing was calculated for each dyad in each direction (i.e., demonstrator-to-observer and vice versa) separately and then compared between different experimental groups. Due to the fact that, during baseline, both demonstrators and observers showed minimal freezing levels, there were not enough freezing time points to calculate the Granger causality for each dyad during this period. Therefore, to examine the effect of familiarity, the analysis was restricted to the shock period (Fig 7A and 7B).

#### Results from the individual familiarity experiment

A MANOVA with Granger causality of both directions as dependent variables and familiarity (0, 1, 3, 5 weeks) as fixed factors revealed no significant effect of familiarity in either direction: demonstrator to observer (F_[3,28]_ = 0.437, *p* = 0.728) and observer to demonstrator (F_[3,28]_ = 0.496, *p* = 0.688), indicating that time spent together as cagemates did not affect the temporal coupling of the freezing of the dyad (Fig 7A).

#### Results from the strain familiarity experiment

A MANOVA with Granger causality of both directions as dependent variables and familiarity (same strain versus different strain) as fixed factors revealed a small effect of condition in the demonstrator-to-observer direction (F_[1,58]_ = 4.726, *p* = 0.034) but not in the observer-to-demonstrator direction (F_[1,58]_ = 0.210, *p* = 0.648). In the demonstrator-to-observer direction, the Granger causality was bigger for same-strain dyads compared with dyads composed of different strains, indicating that there was more information flow from the demonstrator to the observer when both animals were from the same strain than when they were from different strains (Fig 7B).

### The effect of prior shock exposure

All observer rats reported above have been exposed to foot shocks prior to the interaction test because our group has previously shown, using 0.8-mA shocks and female Long-Evans rats, that previous experience with foot shocks is necessary for observers to display robust vicarious freezing [8]. However, because the present study used stronger shocks (1.5 mA) and male rats (which generally show higher absolute levels of freezing compared with the females [44]), we tested how much shock preexposure influences information transfer under these conditions. An additional group of animals were treated as the animals in the 3-week familiarity group described above, except that—during the shock preexposure phase—these observers were placed in the apparatus but not delivered any shocks.

#### Group-level comparisons

**Fig 8:**
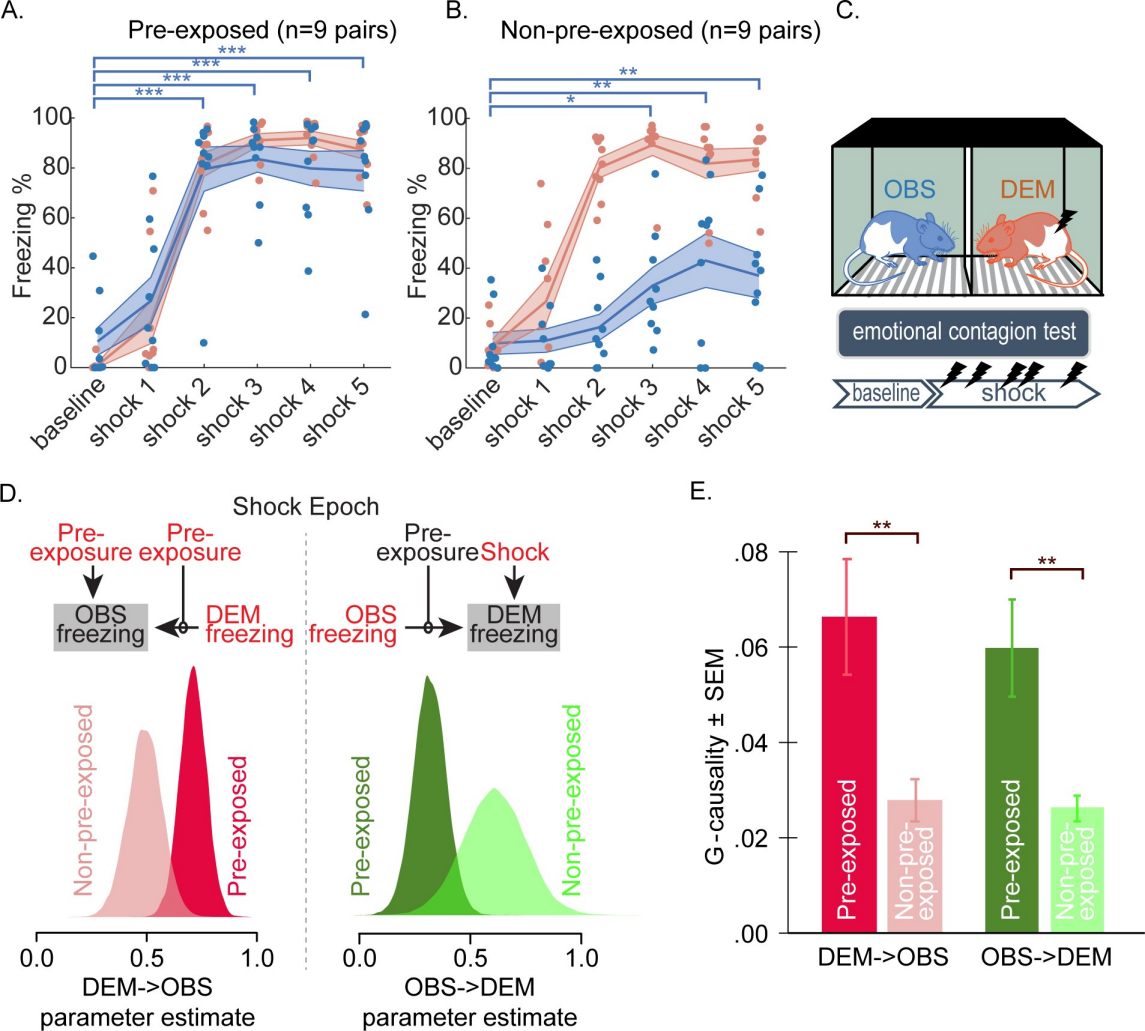
Effect of shock preexposure. (A) Freezing levels for nine preexposed dyads taken from the 3-week group of the individual familiarity experiment for the baseline period and following each of the five shocks. Mean and SEM are shown separately for the demonstrators (orange) and observers (blue). (B) Same for nine other dyads in which the observer rat was not preexposed to shocks prior to the interaction test. For the observers, the significance of one-tailed paired-samples *t* test for freezing following each shock compared with baseline is shown in blue where significant (*:p_unc_ < 0.05, **:p_unc_ < 0.01, ***:p_unc_ < 0.001). Each animal is shown as a dot. (C) Schematic of the interaction test with the two rats color coded as in panels A and B. (D) Parameter estimates from the Bayesian model calculated over the shock period. The variables used for modeling the observer freezing are shown on the left, and those for modeling the demonstrator freezing are shown on the right. Variables that improved the model are shown in red. The effect of preexposure is shown underneath each model as the posterior distribution of the parameter estimates for the connection between DEM → OBS freezing (left) and OBS → DEM (right) separately for the preexposed and nonpreexposed pairs. Note that to improve the estimate for the preexposed animals, here we included all 32 preexposed rats from the individual familiarity experiment (because weeks of familiarity did not influence this parameter significantly), whereas for the nonpreexposed pairs, we have *n* = 9. (E) G-causality F values as a function of direction and preexposure. **: two-tailed *t* test, *p* < 0.01. Data can be found at http://dx.doi.org/10.17632/h8fkyr2z35.1. DEM, demonstrator rat; G-causality, Granger causality; OBS, observer rat; SEM, standard error of the mean.

For these analyses, we compared the dyads with nonpreexposed observers to the dyads from the 3-week individual familiarity experiment group, the observers of which were shock preexposed. Fig 8A and 8B show the percent freezing for baseline and after each of the five shocks. We performed these analyses per shock rather than over the entire shock epoch to visualize the temporal development of freezing.

For the demonstrators’ freezing, a 6 epoch (baseline, shock 1, 2, 3, 4, 5) × 2 preexposure (preexposed versus nonpreexposed) ANOVA indicated a main effect of epoch (F_[2.268,36.294]_ = 165.298, *p* < 0.001) but no preexposure main effect (F_[1,16]_ = 1.570 × 10^−5^, *p* = 0.997) and no epoch × preexposure interaction (F_[2.268,36.294]_ = 1.453, *p* = 0.247). Both groups of demonstrators therefore increased their freezing in response to the shocks, with no strong differences between them. A more detailed look at baseline freezing, however, revealed that the demonstrators from the nonpreexposed batch froze more (mean = 8.7% ± SEM = 2.6%) than those from the preexposed batch (mean = 0.9% ± SEM = 0.8%). If the effect of shocks is quantified as the increase of freezing over the entire shock period minus freezing during baseline (delta = Freezing_dem,shock_ − Freezing_dem,baseline_), then there is a trend for the expected reduction of freezing in demonstrators paired with the nonpreexposed observers that froze less: a *t* test for delta_pre-exposed_ > deltra_nonpreexposed_, t_(16)_ = −1.29, p_1tailed_ = 0.1, p_2tailed_ = 0.2.

For the observers’ freezing, a 6 epoch (baseline, shock 1, 2, 3, 4, 5) × 2 preexposure (preexposed versus nonpreexposed) ANOVA on observer freezing revealed highly significant main effects for epoch (F_[3.406,54.497]_ = 21.682, *p* < 0.001), preexposure (F_[1,16]_ = 35.97, *p* < 0.001), and epoch × preexposure interaction (F_[3.406,54.497]_ = 5.804, *p* = 0.001). To test whether there was evidence for shock observation triggering freezing in both groups separately, we performed separate ANOVAs with 6 epochs for each preexposure group. For the preexposed observers, the epoch main effect was highly significant (F_[2.545,20.359]_ = 21.33, *p* < 0.001). Post hoc uncorrected *t* tests revealed that, compared with baseline, freezing was increased from the second shock onward (for baseline versus shock 2, 3, 4, and 5, t_(8)_ > 6.867, *p* < 0.001, Fig 8A). For the nonpreexposed observers, the main effect of epoch was also significant (F_[2.767,22.139]_ = 4.877, *p* = 0.011). Post hoc uncorrected *t* tests showed that, compared with baseline, freezing was significantly increased from the third shock onward (for baseline versus shock 3, 4, and 5, t_(8)_ > 2.375, *p* < 0.023, Fig 8B). In addition, a *t* test comparing baseline freezing across the two groups revealed no significant difference (t_[16]_ = 0.094, *p* = 0.926). In summary, observers at both preexposure conditions showed some evidence of freezing in response to the demonstrator getting shocks, but this effect was stronger in the preexposed observers.

#### Bayesian model comparison

Bayesian modeling was used to estimate the strength of the connection in the demonstrator-to-observer and observer-to-demonstrator directions and whether these strengths differ across preexposure conditions. We adapted the winning models from the individual familiarity experiment model of Table 1 for this purpose by assuming a modulator of preexposure on the connection between demonstrator to observer and observer to demonstrator and allowing for an effect of preexposure directly onto the observer’s freezing (Fig 8D). We then explored the posterior distributions of the parameter estimates to analyze the effect of preexposure. Similar results were obtained by model comparison (see Table 2). Note that, to fit the parameters for the preexposed group, we used all 32 animals from the individual familiarity experiment here, irrespective of weeks spent together, given that weeks spent together was not found to be a relevant parameter.

**Table 2:** Model comparisons for the preexposure experiment.

**Explaining Demonstrator Freezing in the Preexposure Experiment**				
**Model**	**1**	**3**	**2**	**4**
**Elpd**_**loo**_ **Estimate**	**11.6**	**19.6**	**50.0**	**57.7**
**SE**	**10.6**	**11.7**	**13.7**	**15.5**
**Shock**_**dem**_	---	---	**0.53** (0.44–0.62)	**0.45** **(0.36–0.54)**
**Freezing**_obs_*Shock_**dem**_	**1.12** (1.00–1.24)	---	**0.21** (0.04–0.38)	**---**
**Freezing**_obs_*PreExposure*Shock_**dem**_	---	**1.03** (0.92–1.14)	---	**0.31** **(0.15–0.46)**
**Freezing**_obs_*NoPreExposure*Shock_**dem**_	---	**1.45** (1.31–1.50)	---	**0.89** **(0.54–1.24)**
**Sigma**_**dem**_	**0.21** (0.18–0.24)	**0.19** (0.16–0.22)	**0.12** (0.11–0.15)	**0.11** **(0.09–0.13)**
**Explaining Observer Freezing in the Preexposure Experiment**				
**Model**	**1**	**3**	**2**	**4**
**Elpd**_**loo**_ **estimate**	**13.6**	**23.9**	**26.5**	**30.4**
**SE**	**7.8**	**9.6**	**6.8**	**8.5**
**PreExposure**_**obs**_	---	---	**0.15** (0.09–0.20)	**0.11** **(0.05–0.17)**
**Freezing**_dem_*Shock_**dem**_	**0.73** (0.63–0.82)	---	**0.57** (0.47–0.67)	**---**
**Freezing**_dem_*PreExposure*Shock_**dem**_	---	**0.86** (0.76–0.96)	---	**0.69** **(0.57–0.82)**
**Freezing**_dem_*NoPreExposure*Shock_**dem**_	---	**0.38** (0.22–0.54)	---	**0.38** **(0.24–0.53)**
**Sigma**_**obs**_	**0.20** (0.18–0.24)	**0.18** (0.15–0.21)	**0.17** (0.15–0.20)	**0.16** **(0.14–0.19)**

Separate models were constructed to describe the level of freezing of the demonstrator and the observer. Our main aim was to estimate and interpret the coupling parameter between observer and demonstrator from the winning models of the individual familiarity experiment separately for preexposed and nonpreexposed animals. Fig 8 shows these parameters from model 4 in the table for each case. In addition, here, we also show the performance of that model with regard to models that do not estimate the parameters separately for the two groups (models 1 and 2) or that do not include a shock variable for the demonstrator or a main effect of preexposure on observer freezing. The models are ordered based on their increasing Elpd_loo_, with the worst model on the left and the best model on the right. The first column lists the variables included in each model. Values in the table indicate the parameter estimates with their credible interval below (2.5%–97.5%). The last column in bold always indicates the winning model.

Abbreviations: —, variable not included in that model; dem, demonstrator; Elpd_loo_, expected log pointwise predictive density according to the leave-one-out approximation; obs, observer SE, standard error of the Elpd_loo_

The model explaining the demonstrator’s freezing during the shock period shows that Freezing_dem_ = 0.45 × Shock_dem_ + 0.31 × Freezing_obs_ × Preexposure + 0.89 × Freezing_obs_ × No Preexposure (model 4, Elpd_loo_ estimate = 57.7, SE = 15.5; Table 2A). This shows that when a shock was given, the demonstrator freezes 45% plus 0.31 times the freezing of a preexposed observer or plus 0.89 times the freezing of a nonpreexposed observer. Looking at the distribution of the parameters (Fig 8D right) and credible intervals (Table 2A), we see that both do not overlap with 0, suggesting that how the observer responds to the demonstrator influences the demonstrator’s freezing in both groups. We can also see that the credibility intervals for the two groups do not overlap with each other, suggesting that preexposure influenced this parameter. This is also visible from a model comparison approach in that model 4, which fits separate parameters per group, slightly outperforms model 2, which does not.

During the shock epoch, for the observer, the model shows Freezing_obs_ = 0.11 × Preexposure_obs_ + 0.69 × Freezing_dem_ × Preexposure_obs_ + 0.38 × Freezing_dem_ × No Preexposure (model 4, Elpd_loo_ estimate = 30.4, SE = 8.5, Table 2B). Examining the posterior distribution of the demonstrator-to-observer parameter separately for preexposed and nonpreexposed observers (Fig 8D, left) shows that neither includes 0 in its credibility intervals, showing that the demonstrator’s freezing affects observer freezing in both cases, but the preexposed parameter estimates are higher, with the 95% credibility intervals not overlapping (Table 2). Again, the separation of the parameter estimates across the two groups is also visible from the fact that model 4, which includes one parameter per group, slightly outperforms model 2, which does not.

The Bayesian parameter estimates thus confirm that preexposure shifts the coupling across individuals. Although the coupling is positive in both groups, the coupling in the demonstrator-to-observer direction is reduced when observers lack experience with shocks. This reduction is straightforward to interpret because the overall freezing level of demonstrators is comparable across the two groups. Surprisingly, the parameter estimates in the feedback observer-to-demonstrator direction was higher for nonpreexposed observers. This is counterintuitive at first sight, but one must take into account that, compared with preexposed animals, the observer freezing level was much reduced in the nonpreexposed observers, but the demonstrator freezing level remained relatively unchanged. In both groups, we found a significant feedback in the sense that the posterior distribution shifted away from 0 (Fig 8D, right). This indicates that, even in the nonpreexposed animals, demonstrators paired with observers that froze less also froze less. Given the lower values of freezing in the nonpreexposed observers, this would then necessarily appear as a higher gain parameter.

#### Granger causality

We also examined the effect of preexposure on the interaction between the animals as estimated using Granger causality on the second-by-second freezing during the shock epoch. Fig 8E shows the Granger causality values in both directions as a function of preexposure. Entering the data in a 2 directions (demonstrator-to-observer versus observer-to-demonstrator) × 2 preexposure groups (preexposed versus nonpreexposed) ANOVA reveals no main effect of direction (F_[1,16]_ = 0.56, *p* = 0.47) or direction × preexposure interaction (F_[1,16]_ = 0.18, *p* = 0.67) but a significant main effect of preexposure (F_[1,16]_ = 11.82, *p* = 0.003), indicating that the coupling was altered in both directions. Finally, to examine whether Granger causality values were significant even in the nonpreexposed animals, as in the section Moment-to-moment coupling—Granger causality for the preexposed animals, we calculated a single Granger model over all the nonpreexposed pairs during the shock period. This evidenced significant Granger causality in both directions (demonstrator-to-observer, Granger causality F = 0.0027, *p* = 0.02; observer-to-demonstrator, Granger causality F = 0.0032, *p* = 0.004; Granger causality order 19 as automatically detected), which was numerically larger in the observer-to-demonstrator direction.

### Role of the ACC in information transfer

Given that both model comparison and Granger causality suggest that the behavior of the observer feeds back on the behavior of the demonstrator, we wanted to experimentally probe this feedback by reducing the freezing reaction of the observer and testing whether that would reduce freezing in the demonstrator. In humans, the ACC has been considered one of the core regions activated by witnessing the pain of others [2,5,45]. This region has its homolog in the ACC of the rat [46] and has been implicated in emotional contagion and empathy in rodents as well [11,13,30,39,41,47–49]. We therefore predicted that deactivating this region in observers should reduce their vicarious freezing and, by virtue of the feedback connection that the individual and strain familiarity experiments suggest, reduce the freezing of the demonstrator. To examine this possibility and confirm the role of the ACC in social information transfer in rodents, a fourth experiment was conducted in which the ACC of the observers was deactivated using muscimol, and the impact on vicarious freezing was studied in both observers and demonstrators (this condition is also part of a larger experiment reported in [41]).

**Fig 9:**
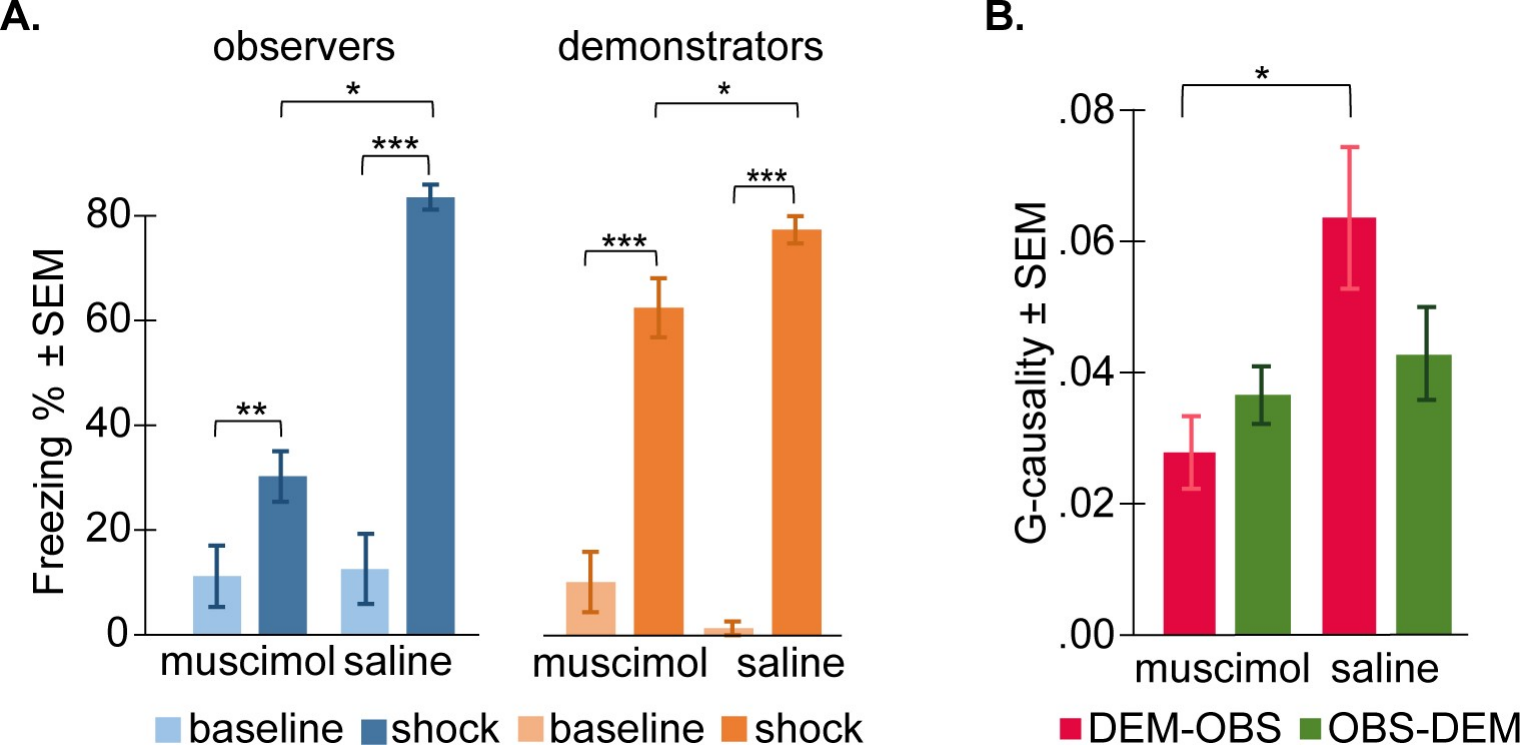
The role of ACC. (A) Effect of ACC deactivation on freezing. Percentage of time the observers (in orange) and demonstrators (in blue) spent freezing during baseline (light color) and the shock period (dark color) after ACC deactivation (muscimol) or after control treatment (saline). Freezing percent = 100*freezing time/total time of the corresponding period. (B) Effect of ACC deactivation on the flow of information. Mean ± SEM of the G-causality values in the demonstrator-to-observer direction (DEM–OBS, in red) and in the observer-to-demonstrator direction (OBS–DEM, in green) during the shock period, after ACC deactivation (muscimol), or after control treatment (saline). Data can be found at http://dx.doi.org/10.17632/h8fkyr2z35.1. ACC, anterior cingulate cortex; DEM, demonstrator rat; G-causality, Granger causality; OBS, observer rat; SEM, standard error of the mean.

To test the effect of muscimol on the observer freezing, a 2 period (baseline versus shock) × 2 condition (muscimol versus saline) ANOVA was conducted on percent freezing scores to test the effect of ACC deactivation on socially triggered freezing of the observers (Fig 9A). All observers froze significantly more during the shock period (muscimol: mean ± SD = 30.24% ± 11.77%; saline: mean ± SD = 83.57% ± 6.79%) than during the baseline (muscimol: mean ± SD = 11.21% ± 14.35%; saline: mean ± SD = 12.60% ± 18.87%), as confirmed by the significant main effect of period (F_[1,12]_ = 126.556, *p* < 0.0001). Paired-samples *t* tests confirmed that, in both conditions, the observers’ freezing levels were significantly higher during the shock period compared with the baseline (muscimol: t_[5]_ = 5.617, *p* < 0.005; control: t_[7]_ = 11.101, *p* < 0.0001), showing socially triggered freezing in both ACC-deactivated and control observers. However, observers with muscimol injections froze significantly less compared with saline controls (F_[1,13]_ = 100.805, *p* < 0.0001), indicating that ACC is necessary for full-fledged socially triggered freezing. A significant period × condition interaction effect was also found (F_[1,13]_ = 31.737, *p* < 0.0001), reflecting that the impact of muscimol was larger during the shock period. To explore whether muscimol may have altered locomotion more generally, we also used quantified activity during the baseline and shock period for both groups of injected animals (i.e., observers). Activity is expressed as the average percentage of pixels that changed within a 100-ms period. At baseline, the groups did not differ significantly in activity (muscimol: mean ± SD = 2.9 ± 1.0; saline: mean ± SD = 3.6 ± 1.3; t_[12]_ = 1.25, *p* = 0.23), but during the shock observation period, they did (muscimol: mean ± SD = 1.1 ± 0.5; saline: mean ± SD = 0.1 ± 0.04; t_[12]_ = 5.1, *p* < 0.001). That muscimol increased activity during the shock period echoes that muscimol reduced freezing. Indeed, a 2 period (baseline versus shock) × 2 condition (muscimol versus saline) ANOVA on the tracked motion confirmed the effect of muscimol found in the freezing data: main effect of condition was not significant (F_[1,12]_ = 0.06, *p* = 0.8), but the main effect of period (F_[1,12]_ = 49, *p* < 0.001) and the period × condition interaction (F_[1,12]_ = 8.1, *p* < 0.015) were significant.

To test our hypothesis that demonstrators paired with muscimol observers would show reduced freezing compared with those paired with saline observers, a one-tailed *t* test was performed on demonstrator’s freezing during the shock period, and results were significant (t_[12]_ = 2.397, *p* < 0.024, Fig 9A). An ANOVA including 2 conditions (muscimol versus saline) × 2 periods (baseline versus shock) confirmed this effect as a significant interaction (F_[1,12]_ = 19.837, *p* < 0.001), with the effect of condition larger during the shock than baseline period.

To further investigate the impact of ACC deactivation on the temporal coupling across the animals, a Granger analysis was performed on the second-to-second freezing of the observers and the demonstrators (Fig 9B). It was expected that deactivating the ACC of the observer should perturb the information transfer from the demonstrator to the observer because a structure necessary for triggering vicarious freezing in the observer (i.e., ACC) would be impaired. It was also expected that the transfer in the observer-to-demonstrator direction should remain unaffected because the brain of the demonstrator was not injected with muscimol. To compare differences between the two groups, a MANOVA with Granger causality of each dyad in both directions (demonstrator-to-observer and observer-to-demonstrator) as dependent variables and conditions (muscimol versus saline) as fixed factors was conducted. A significant effect of condition in the demonstrator-to-observer direction (F_[1,12]_ = 6.620, *p* = 0.024) was found, but not in the observer-to-demonstrator direction (F_[1,12]_ = 0.424, *p* = 0.527). In the demonstrator-to-observer direction, the Granger causality was significantly smaller for the ACC-deactivated group compared with control dyads, indicating that the observers’ freezing responses were less influenced by the demonstrators’ when the observers’ ACCs were deactivated and that the temporal dynamic within the dyad was impaired by the manipulation.

**Fig 10:**
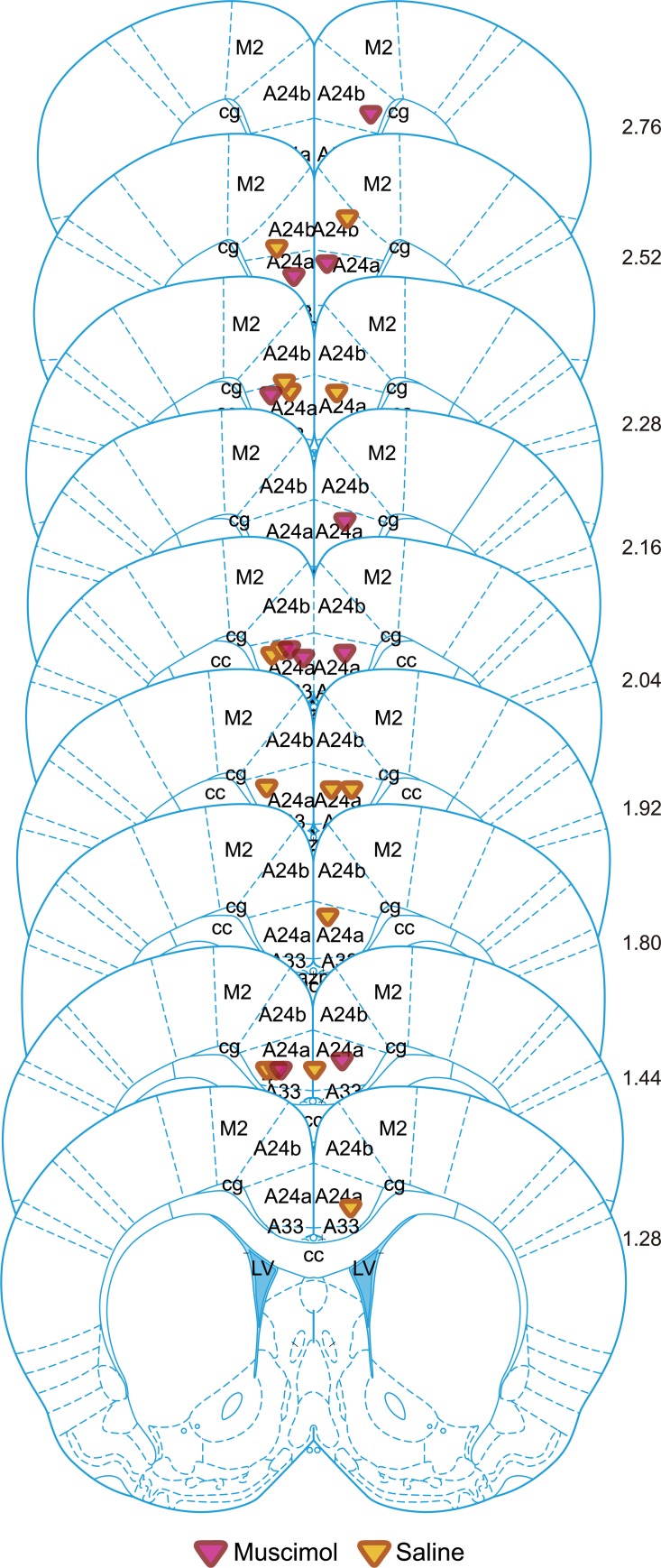
Localization of the cannula tips on coronal sections. Coordinates estimated based on Nissl stains. A24 and A33 refer to area 24 and area 33, as in the atlas and the work of Vogt [46,50]. Data can be found at http://dx.doi.org/10.17632/h8fkyr2z35.1. cc, corpus callosum; cg, cingulate gyrus; LV, lateral ventricle.

Histological reconstructions confirmed that we successfully targeted ACC, particularly region 24a and 24b (Fig 10).

### Signal detection perspective

**Fig 11:**
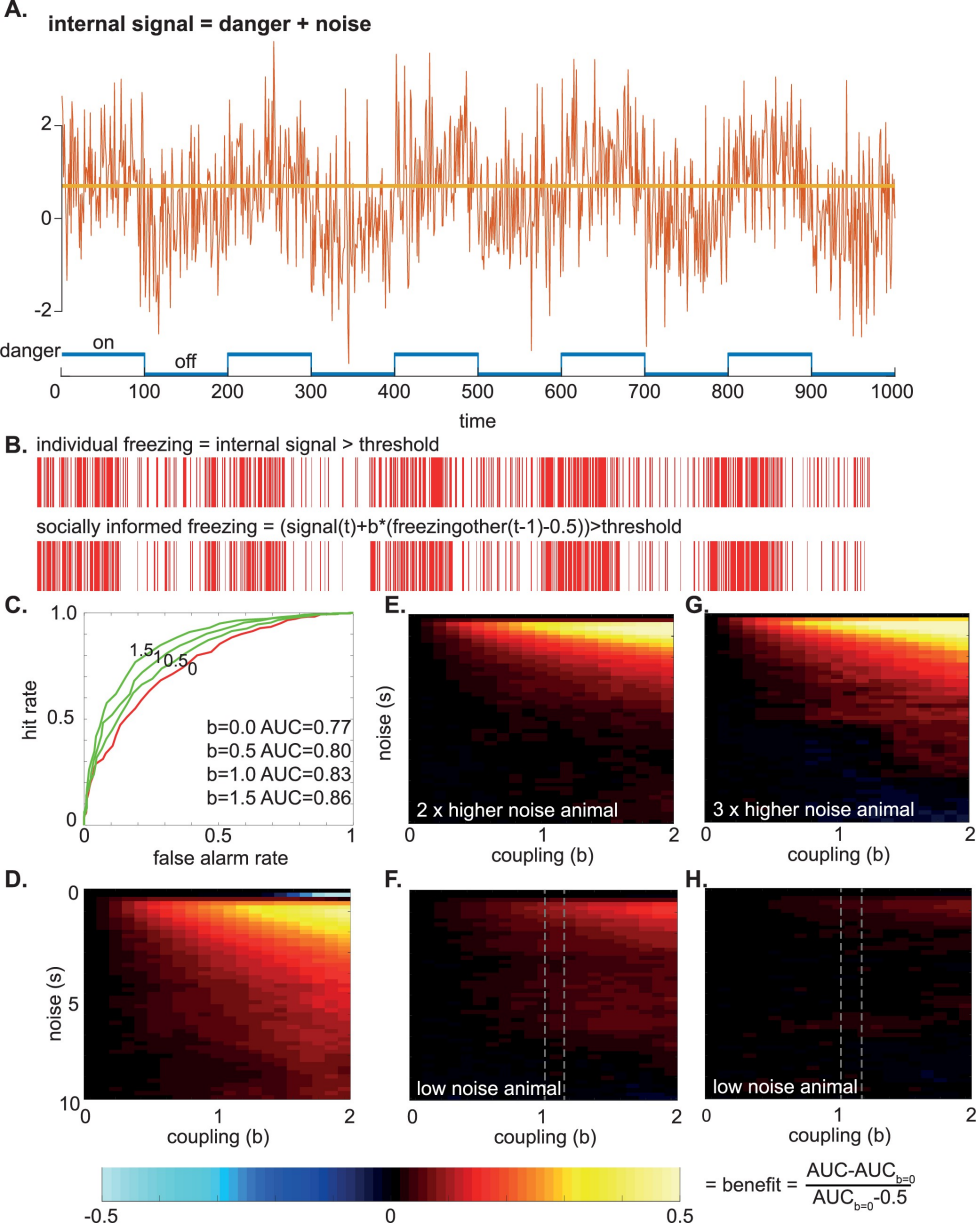
Computational modeling of danger detection. (A) Internal danger signal simulated for an animal that is exposed to 100 time points of danger and 100 time points of no danger with noise added. The animal freezes when the danger signal surpassed a certain threshold (yellow line). (B) Time series of freezing for an animal by itself (individual freezing) or for one that is additionally taking the freezing of another animal into account (socially informed freezing). (C) Accuracy of danger detection shown as the area under the ROC curve (AUC) for different coupling factors (b = 0–1.5). A higher coupling factor increases the AUC. (D) Benefit of taking the freezing of others into account when both animals have the same access to danger signals, i.e., experience the same noise level. (E-H) Animals with twice (E) or thrice (G) as much noise as compared with another animal ([F] and [H], respectively) had stronger benefits from coupling. However, the low-noise animals (F, H) experience no disadvantages. The dotted lines indicate the coupling regime that our animals appeared to be in the individual and strain familiarity experiments. Codes can be found at http://dx.doi.org/10.17632/h8fkyr2z35.1. AUC, area under the curve; ROC, receiver operating characteristic.

We designed simulations that explore whether, in the presence of uncertainty, including the behavioral reaction of others, the accuracy of danger signal detection can improve. Several simulations were performed that compared danger detection performance of individuals with or without social information (i.e., taking or not taking the freezing from another animal into account) and with equal or unequal access to the danger signals (see Materials and methods for details). Briefly, the logic of the simulations is that a danger signal is turned on and off over time (blue in Fig 11A), generating an internal danger signal in the animal after addition of noise of magnitude σ. In the individual condition, the animal then decides whether to freeze or not to freeze based on whether the internal signal surpasses a threshold (yellow in Fig 11A), leading to a time series of freezing decisions (red in Fig 11A and time series shown in Fig 11B). In the social simulation, the individual additionally takes into account the freezing at time *t* − 1 of the other animal in deciding whether to freeze at t, by adding b*(Freezing_other[t − 1]_ − 0.5) to its internal danger signal (Fig 11B).

When both animals have the same access to danger signals (i.e., experience the same signal-to-noise ratio), the decision to freeze becomes more accurate if animals take the freezing of the other animal into account. Fig 11C illustrates this phenomenon at a relatively low noise level (σ = 1). If the animal does not take the freezing of the other into account (coupling b = 0, red curve), the area under the red receiving operating characteristic curve (area under the curve [AUC]) is equal to 0.77. Increasingly taking the freezing of the other into account (b from 0.5 to 1.5) augments the AUC, meaning a more accurate danger detection. This benefit in danger detection can be seen as comparatively more freezing (Fig 11B, red) when danger is present and less when it is absent for the socially informed freezing. This means that animals that are influenced by the freezing of the other will freeze more when there is danger and freeze less when there is none. Repeating this analysis for different noise levels (σ) and coupling (b) reveals that over a wide range of parameters, there are either benefits (red and yellow colors) or no disadvantages (black, Fig 11D). Only in very specific cases (very low noise σ < 0.5 and high coupling b > 1) is there a loss of performance (Fig 11D). Analyzing the time series shows that these rare cases occur when the animal that is no longer in danger (time t) erroneously persists in its freezing because the other animal was freezing at *t* − 1.

In our experiments, one animal, however, has privileged access to danger signals because it experiences the shock itself, whereas the other has less direct access. What was surprising is that the more-informed demonstrators still relied on the behavior of the less-informed observers. To examine such scenarios, additional models were simulated to capture unequal access to danger signals. This was done by imposing twice or thrice as much noise on one animal compared with the other. In these models, the animal with more noise has stronger benefits from coupling (Fig 11E and 11G); however, the other animal experiences no disadvantages (no cold color in Fig 11H) and sometimes even reaped advantages from taking the less-informed animal’s freezing into account (warm colors in Fig 11F).

One may wonder how these coupling parameters compare with those we found in our Bayesian models for the demonstrators. In our simulation, b represents the ratio of the social/direct danger signal. Accordingly, in our Bayesian models for demonstrator freezing (Table 1), it can be approximated as the fraction Freezing_obs_/Shock_dem_ and would have the value b = 0,79 and b = 1,33 (gray dotted lines in Fig 11F and 11H) for the individual and strain familiarity experiments, respectively. In summary, we find that moderate coupling in the order of magnitude found in our Bayesian modeling (b is approximately equal to 1) always improved the decision-making of our simulated animals.

## Discussion

Our aim was to develop a methodology to quantify bidirectional information transfer across rats in the context of a potential danger. We combined a paradigm initially developed to investigate emotional contagion with analysis techniques that quantify information transfer in each direction. We asked whether information transfer is bidirectional, whether it depends on familiarity and prior experience, and how ACC deactivation influences information transfer.

Bayesian modeling revealed that there was information transfer not only from the demonstrator to the observer but also from the observer to the demonstrator. This was even the case across unfamiliar individuals and unfamiliar strains. Granger causality analyses further confirmed temporal coupling between the demonstrator and the observer in both directions. To our knowledge, this is the first rigorous quantitative demonstration of bidirectional social information transfer in the now widely used rodent emotional contagion paradigms and provides a better fit to the data than the traditional one-way focus of current studies. Conceiving of the influence as mutual has the conceptual advantage of integrating what has been considered emotional contagion and social buffering (Fig 1) as two sides of information transfer, thereby providing a unifying framework across related fields that have so far engaged in relatively little cross talk.

In a smaller experiment, we tested a group of observers without preexposure to foot shocks. We found that, compared with the animals from the main experiment that had been preexposed to shocks, omitting preexposure led to a significant reduction in observer freezing and interfered with the coupling between the animals. This confirms our earlier finding in female rats that preexposure boosts information transfer [8]. Some, albeit weaker, freezing and coupling was, however, observed also in pairs with naïve observers, which dovetails with the mice literature that has often studied freezing in naïve observers [12,13,51]. Overall, this effect of preexposure suggests that information transfer about danger is not entirely inborn. Instead, part of the information transfer depends on some form of learning, as has been emphasized in the eavesdropping literature that explores how danger information is transferred across members of different species [52–54]. During preexposure, the observers repeatedly experience a systematic coupling between (1) their own pain, fear, and freezing and (2) the sound of their own pain squeaks and the conspicuous silence accompanying freezing [55]. This could lead to Hebbian [56,57] reinforcement of the synapses connecting the sound of squeaking and cessation of movement to the observer’s own pain, fear, and defensive neurons in the ACC and amygdala [39,41]. As a result, hearing the behavior of the demonstrator would then more strongly trigger neurons involved in matching emotional states and behavior in the observer [39,41] and, thereby, increase the behavioral coupling across them. A less-selective explanation of the effect of prior exposure is that preexposure primes rats to expect danger and biases them to respond to a wider gamut of stimuli with freezing. Comparing naïve observers and observers preexposed to foot shock against a third group of observers preexposed to a different stressor (e.g., a forced cold water swim challenge [58]), could, in the future, help disentangle these alternative mechanisms. In mice, results indicate that prior exposure to forced swimming, unlike prior exposure to foot shocks, does not sensitize mice to freeze while witnessing another mice receive foot shocks [14], speaking in favor of a more selective explanation, but whether the same is true in rats remains to be investigated.

In terms of neural mechanisms, we show that ACC is crucially involved in this information transfer. More precisely, temporarily deactivating this brain structure in one member of the social interaction attenuates the information transfer to the injected individual. Furthermore, this deficit feeds back and influences the behavior of the brain-intact partner, showing again bidirectional information transfer.

Our results show that rats do show information transfer, even across unfamiliar individuals. We also find comparatively little evidence that the information transfer is increased in more-familiar animals. The Bayesian model fitting shows that the parameter estimates for the transmission is similar across the different familiarity levels, with largely overlapping distributions. The Bayesian model comparison further shows that models that stratify the connection based on familiarity do not outperform models that assume the same strength of connections for all groups. These models, however, were calculated based on the overall level of freezing in the entire 12-minute period. It is possible that the effect of familiarity is more evident in a fine-grained analysis of the second-to-second decision to freeze. However, in the individual familiarity experiment, such a fine-grained Granger causality analysis also evidenced no effect of familiarity while confirming a significant bidirectional coupling across all dyads. Dyads that saw each other for the first time on the day of testing coordinated their freezing as closely as those that had spent 5 weeks together. Only toward extreme strangers—i.e., animals of a strain they had never encountered before—did that analysis reveal a small decrease of Granger causality, and then only in the demonstrator-to-observer direction. In other words, although observers will respond to the shock given to the demonstrator of an unknown strain (with levels of freezing similar to those when witnessing their own strain, as revealed by the Bayesian modeling), the moment at which they will show that reaction is slightly less tightly linked to that of the demonstrator compared with animals from the same strain.

The relative lack of familiarity effect we find in rats is different from findings in mice, in which information transfer seems to depend more strongly on familiarity [10,11,32,33]. The difference in social structure between mice and rats may account for part of this difference [59]. Male mice do not tolerate other mature males around them, and the presence of other mature individuals triggers a strong glucocorticoid-mediated stress reaction that inhibits information transfer [33]. Rats, on the other hand, live in much larger groups with other adult males that are tolerated [59]. It may be that, in that structure, seeing an unfamiliar individual does not produce the kind of stress response that would shut down information transfer and, thereby, allows the significant transfer we document in our design. An alternative explanation is that the rats showed information transfer in all cases because they failed to recognize the difference between familiar and unfamiliar partners. However, this cannot account for our data because our control experiment (Fig 6) demonstrates that the rats can perceive the difference between familiar and unfamiliar individuals at the illumination levels used during our paradigm. Finally, we must also consider the possibility that freezing in our observers was so strong that a ceiling effect prevented us from witnessing the effect of familiarity. Future experiments in which weaker shocks are given to the demonstrators might help address this possibility. However, when investigating only Sprague Dawley observers, which show more moderate freezing, we still see that a model not including a same/different strain modulator (Elpd_loo_ = 34.1) outperforms one that does (Elpd_loo_ = 32.9).

So far, we have looked at the coupling of the freezing behavior across two rats agnostically as a form of information transfer in the context of danger. This begs two important questions. First, which modality conveys the information? In rats, there is evidence that both fear-induced 22-kHz ultrasonic vocalizations [60] and the audible silence triggered by freezing are stimuli that can trigger freezing in shock-preexposed listeners [55], and we have shown that pain squeaks are another likely channel [41]. Testing rats in complete darkness shows that visual cues seem not to be necessary [55]. In mice, visual information was found to be the only critical factor in social transmission of acetic acid–induced pain [32], whereas it is effective, but not sufficient, in conveying foot shock–induced pain information to others [11]. Furthermore, olfactory cues are necessary and sufficient for social buffering in rats [61]. Taken together, the modality through which the affective state is transferred from one animal to another might differ depending on species and paradigms. More work will be needed to systematically identify the stimulus features coupling the two animals, and it is likely that the coupling is the result of a flexible integration of different features. An exciting question for future research might also be to identify which of these modalities might depend on preexposure to shocks (e.g., the sound of freezing or pain squeaks) and which might not (e.g., pheromones).

Second, what is being transferred? One important distinction relates to the distinction between behavioral mimicry and emotional contagion [30,31]. The former refers to cases in which a behavior in one animal triggers a similar behavior in another, and the latter refers to cases in which an affective state in one animal triggers a similar affective state in another. By focusing on the coupling of freezing across rats in our analysis, we demonstrate behavioral mimicry in which the level of freezing aligns across animals. Whether we also have an alignment of affective state, and whether such an affective alignment causes or results from the behavioral mimicry, remains unclear. As James would put it: Does the observer freeze because the squeaking and freezing of the demonstrator scared him or is the observer scared because he froze [62]? Future experiments recording a wider gamut of affective indicators (e.g., heart rate, startle potentiation, pupil dilation, ultrasonic vocalizations, and pheromones) could shed light on these questions. However, procedures similar to ours not only trigger freezing while interacting with the partner but also induce fear learning: one can observe freezing when the observer is placed in the test context alone 48 hours later and when exposed to a sound coupled with shocks to the demonstrator 24–48 hours later [63,64]. Additionally, using electrophysiological recordings in a similar paradigm, we found that a significant proportion of neurons in the observer’s ACC that respond while the observer is in pain also respond within 100 ms of the demonstrator receiving a shock [65]. This region of the ACC has been associated with the affective dimension of pain [66], suggesting an affective alignment of the observer to the affective pain state of the demonstrator.

Because many have looked at the social transmission of freezing in mice and rats as an example of empathy [30,51], and empathy has often been tightly associated with prosocial motivations [29,30], it might be tempting to look at the information transfer we document as suggesting that observers freeze because they are scared on behalf of the demonstrator. The bidirectional information flow we demonstrate, the weak effects of familiarity, and our computer simulations invite us to look at the social transmission of freezing from a more selfish point of view. Our simplified simulations show that the accuracy of danger detection in a noisy environment is improved if an animal takes the freezing behavior of other animals into account. Importantly, in the parameter range that we find in our Bayesian modeling, in which demonstrators give similar weights to the shock and social information, we found that taking social information into account never decreases the danger detection performance of the simulated individuals in a group. Although these simulations have many limitations, in particular the fact that they assume that noise is independent across animals, they encourage us to look at the coupling of freezing from a different perspective. The increase in freezing in observers witnessing the pain of a demonstrator and the reduction in freezing of demonstrators paired with less freezing observers could simply be mechanisms for anticipatory defensive behavior when a danger is present but not when it is absent, respectively. Picking up the freezing of others becomes akin to using others as antennas to amplify often noisy danger signals.

In this interpretation, the increase in freezing often associated with emotional contagion (Fig 1A) then occurs when the behavior of another animal signals higher danger, as is the case when a calm observer suddenly perceives nocifensive behavior in a demonstrator, whereas the decrease in freezing often associated with social buffering (Fig 1B) happens when the behavior of another animals indicates lower danger, as is the case when a distressed demonstrator perceives a seemingly calmer observer. At a group level, the dyad then comes to a consensus on the level of freezing, something that improves decision-making and has motivated the field of crowd decision-making [67]. This perspective does not imply that coupling of affective states cannot serve altruism but, rather, that it may have evolved under the benefits of a crowd computation of danger, which needs not to be gated by familiarity. Traditionally, eavesdropping, the fact that some mammals and birds show signs of fear when they perceive the alarm calls of other species [52], has been conceived of as different from emotional contagion across individuals of the same species. The former (which has to our knowledge received little attention in rats) is considered a selfish form of information gathering, whereas the latter has often been seen as more prosocial. Our data invite us to consider that they may not be as different after all and to investigate how transmission is progressively altered as the observer and demonstrator are from increasingly dissimilar species.

## Materials and methods

### Ethics statement

In compliance with Dutch and European law and institutional regulations, all experimental procedures were preapproved by the Centrale Commissie Dierproeven of the Netherlands (AVD801002015105) and/or by the welfare body of the Netherlands Institute for Neuroscience (IVD, protocol numbers NIN1493, NIN151101, NIN151104, and NIN181109) in accordance with the Experiments on Animals Act (WOD) with its amendment on 18 December 2014 and EU directive 2010/63/EU.

The humane end points were as follows: The first end point was insufficient recovery after surgery—considered if the animal showed permanent weight loss (more than 15% of the weight immediately after surgery for more than 10 days). The second end point was infection—although we always perform the surgeries in sterile conditions, there was a small possibility of infection around the wound area. Visible signs of pathogenesis were monitored. The following were considered as signs of an unhealthy state of the animal: aberrant behavior, shock, dehydration, weight loss, nose and mouth discharge, bleeding, fits/seizures, and diarrhea. The third end point was if the implant was lost or became loose beyond repair.

All animals recovered well from the surgery and showed no signs of infection, and the probes were firmly in place until the end of the experiment (i.e., no animal was sacrificed because of a humane end point criteria 1 and 2). One animal lost its implant before testing, which resulted in bleeding around the wound; thus, it was euthanized using CO_2_ (see below).

After experiments, all animals in experiments 1, 2, and 3 and the demonstrators in experiment 4 were euthanized by CO_2_ inhalation, starting with 40% O_2_ mixed with 60% CO_2_, until animals were in deep sleep, and then we switched to 100% CO_2_ for at least 15 minutes until no breathing or heartbeats were detected. For experiment 4, which required histological examination and perfusion, observer animals were given an overdose of sodium pentobarbital (90 mg/kg, ip), and depth of anesthesia was verified (by a lack of rear foot reflexes and low respiration rate) prior to perfusion.

### Subjects

The following rats were obtained from Janvier Labs (France): for experiment 1 (i.e., individual familiarity), 80 male Long-Evans rats (6–8 weeks old); for experiment 2 (i.e., strain familiarity), 140 male rats (78 Long-Evans and 62 Sprague Dawley, 6–8 weeks old); for experiment 3 (i.e., preexposure effect), nine male Long-Evans rats; and for experiment 4 (i.e., deactivation of the ACC, also reported in [41]), 36 male Long-Evans. For experiments 1, 2, and 3, upon arrival, animals were housed in groups of four or five in type IV cages with wooden block toys and hiding shelter. For experiment 4, animals were housed in dyads (except for a short period after surgery) in type III cages with wooden block toys. Only animals from the same strain were housed in the same cage. All animals were maintained at ambient room temperature (22–24 ^o^C, 55% relative humidity, SPF, on a reversed 12:12 light-dark cycle, lights off at 07:00) and allowed to acclimate to the colony room for 7 days. Food and water were provided ad libitum.

### Setup

All tests were conducted in a two-chamber apparatus (each chamber L: 24 cm × W: 25 m × H: 34 cm; Med Associates, Fairfax, Vermont, United States). Each chamber consisted of transparent plexiglass walls and stainless-steel grid rods. The compartments were divided by a transparent perforated plexiglass separation, which allowed animals in both chambers to see, smell, touch, and hear each other. For shock preexposure of observers and for the interaction tests, one of the chambers was electrically connected to a stimulus scrambler (ENV-414S, Med Associates). For video recording of the rats’ behaviors, a Basler GigE camera (acA1300-60gm) was mounted on top of the apparatus controlled by EthoVision XT (Noldus, the Netherlands).

### Experimental procedures

All experimental procedures were conducted during the dark part of the animals’ daylight cycle. Fig 2 illustrates the general procedures used for those experiments, except that in experiment 3, no shocks were delivered during preexposure.

#### Experiment 1—Individual familiarity

Observer–demonstrator dyads were randomly allocated to one of the following groups: unfamiliar condition (*n* = 10 dyads), familiar for 1 week (*n* = 10 dyads), familiar for 3 weeks (*n* = 10 dyads), or familiar for 5 weeks (*n* = 10 dyads). A total of 8 dyads were excluded because of technical failure (3 dyads excluded from unfamiliar condition, 1 dyad excluded from the 3 weeks’ familiar condition, and 4 dyads excluded from the 5 weeks’ familiar condition).

Prior to the start of the experimental procedures, animals were randomly paired and assigned the role of observer or demonstrator. Depending on the familiarity condition, observer–demonstrator dyads were housed for 1, 3, or 5 weeks prior to testing. For the unfamiliar condition, 3 weeks prior to test day, animals were housed in dyads of the same role (i.e., either two observers or two demonstrators in one cage). At 10 days prior to the interaction test, all animals were handled every other day for 3 minutes. To habituate animals to the testing conditions, 4 days preceding testing, animal dyads were transported and placed in the testing apparatus for 20 minutes/day for three consecutive daily sessions. The testing apparatus was cleaned with lemon-scented dishwashing soap and 70% alcohol in between each dyad.

To enhance the response to the distress of the demonstrators [8], observer animals experienced a shock preexposure session the day prior to test day. The shock preexposure was conducted in one of the chambers of the test apparatus. To prevent contextual fear, the walls of the chamber were coated with black and white–striped paper, the background music was turned off, the apparatus was illuminated with bright white light, and the chamber was cleaned with rose-scented dishwashing soap and vanilla aroma drops. Observers were individually placed in the apparatus and after a 10-minute baseline, four foot shocks (each: 0.8 mA, 1 second long, random ISI of 240–360 seconds) were delivered. After the shock preexposure session, animals were placed for 1 hour in a neutral cage prior to returning to their home cage.

The testing setup was illuminated with dim red light and cleaned using a lemon-scented dishwashing soap followed by 70% alcohol, and background radio music was turned on. Each observer–demonstrator dyad was transported to the testing room, and animals were placed in the corresponding chamber of the testing apparatus. For the unfamiliar condition, randomly chosen observers and demonstrators from different cages were used to create the testing dyads. For this condition, observers and demonstrators never had contact with each other until test start. For the familiar conditions, observers and demonstrators were from the same cage. The testing order was fully randomized. For all dyads, following a 12-minute baseline, the demonstrators experienced five foot shocks (each: 1.5 mA, 1 second long, ISI of 120 or 180 seconds). Following the last shock, dyads were left in the apparatus for 2 additional minutes prior to returning to their home cage.

#### Experiment 2—Strain familiarity

Observer–demonstrator dyads were randomly allocated to one of four groups in which the demonstrators received foot shocks in the interaction test. The experimental groups consisted of (1) dyads of two Long-Evans (LE-LE, *n* = 23 dyads), (2) dyads of two Sprague Dawleys (SD-SD, *n* = 15 dyads), (3) dyads of a Long-Evans observer and a Sprague Dawley demonstrator (LE-SD, *n* = 21 dyads), or (4) dyads of a Sprague Dawley observer and a Long-Evans demonstrator (SD-LE, *n* = 11 dyads). A total of 10 dyads were excluded because of technical failure (4 dyads excluded from the LE-LE condition, 2 dyads excluded from the SD-SD condition, and 4 dyads excluded from the LE-SD condition).

Upon arrival, all animals were randomly paired in same-strain and same-role dyads (i.e., each dyad of animals was assigned the role of either observer or demonstrator), which were pair-housed together. Handling and habituation procedures were conducted in the same way as in experiment 1, with the exception that the shock preexposure was conducted following the first habituation, and this was followed by the second and third habituation sessions. In addition, during habituation, a white plastic perforated floor was added on top of the grid floor of the observer’s chamber.

The shock preexposure for all animals was conducted following the first habituation session. The shock preexposure parameters were identical to those described for experiment 1.

The testing procedures and parameters for experiment 2 were the same as those described for the unfamiliar condition of experiment 1. Observers and demonstrators were randomly chosen according to the experimental condition (e.g., for the SD-LE condition, a Sprague Dawley from an observer cage and a Long-Evans from a demonstrator cage were selected). Although all animals were kept in the same room during acclimation, observers and demonstrators did not have contact with each other (nor to any individual of a different strain) until the start of the test. Similar to habituation, a white perforated plastic was placed on top of the grid floor of the observer’s chamber.

#### Experiment 3—Preexposure effect

The nonpreexposed group consisted of observer–demonstrator dyads (*n* = 9 dyads) that were housed together for 3 weeks prior to the test. Handling and habituation were conducted exactly as described for experiment 1.

Observer–demonstrator dyads in the nonpreexposed group were placed in the apparatus as described for experiment 1 but did not receive any foot shocks during the shock preexposure session. Afterward, animals were placed for 1 hour in a neutral cage prior to return to their home cage.

The nonpreexposed group was tested with exactly the same procedures and parameters as described for experiment 1.

#### Experiment 4—ACC deactivation

The shock observation condition with ACC deactivation reported here is part of a larger experiment reported in [41].

Observer–demonstrator dyads were randomly allocated to one of two groups: saline control group (*n* = 10 dyads) or muscimol group (*n* = 8 dyads). Four dyads (two from the control group and two from the muscimol group) were excluded after histological examination suggesting damage of corpus callosum due to injection.

Upon arrival, all animals were randomly housed in dyads, one assigned as the observer and one as the demonstrator.

Cannulas were implanted into ACC, targeting area 24, 1 week prior to behavioral testing (hit: *n* = 14, miss: *n* = 4). To reduce the discomforting effects of surgery, a subcutaneous injection of 0.01–0.05 mg/kg of buprenorphine was administered 30 minutes prior to surgery. All animals were anesthetized using isoflurane (4% for induction and 1%–1.5% for maintenance). The animals were then positioned in a stereotaxic frame with blunt-tipped ear bars, and a midline incision was made. The incision area was cleaned with alcohol and betadine and sprayed with 10% Xylocaine (lidocaine, local anesthetic). Six burr holes were drilled (two for anchoring screws and one for the cannula per hemisphere). Two single guide cannulas (62001, RWD Life Science, San Diego, California, US) were implanted targeting bilateral ACC (AP = +1.7, ML = ±1.6, DV = +1.8 mm) with a 20° angle from the surface of the skull [50] and chronically attached in the observer animals with a thin layer of acrylic cement (Super-Bond C & B, Sun Medical, Shiga, Japan) and thick layers of acrylic cement (Simplex Rapid, Kemdent Works, Swindon, Wiltshire, United Kingdom). To prevent clogging of the guide cannula, a dummy cannula (62101; RWD Life Science) was inserted and secured until the microinjection was administered. After the surgery, an analgesic/anti-inflammatory drug was delivered for pain relief (Metacam, 1 mg/kg, sc) and 0.5 ml of saline sc was given for rehydration. To prevent damage to the cannula, observers were individually housed for 2–3 days in type III cages with wooden block toys during recovery, and then they were socially housed with the previous cagemates. To monitor any possibility of discomfort or pain and to make sure that the animals were having a proper recovery process, the appearance, behavior, state of the incision (wound healing), recovery process, and weight were monitored daily for 3 days and once a week thereafter. Specifically, we scored (0–3, 0 = normal, 3 = highly abnormal) each one of these categories daily for the first 3 days after surgery and then once a week until the end of the experiment. All animals had a total score of 0 in all categories except weight, in which few animals had a score of 1/day for a maximum of 3 days (indicating that they lost some weight, which they quickly recovered). In addition, 24 hours after the surgery, all animals showed normal behavior (i.e., no signs of pain or discomfort or any other abnormality), prompt recovery, and healthy wound healing. This was maintained until the end of the experiment. Animals were allowed to recuperate for at least 7 days prior to test start. After a week of recovery, observers were habituated to fake microinjections and to the experimental setup with their demonstrators for 20 minutes.

Habituation procedures were conducted in the same way as for experiment 1 and 2, except that prior to transport to the experimental room, the observer animals were habituated to a sham infusion procedure.

At 15 minutes prior to the interaction test, observer animals were lightly restrained, the stylet was removed, and an injection cannula (62201, RWD Life Science) extending 0.8 mm below the guide cannula was inserted. Muscimol (0.1 μg/μl) or saline (0.9%) was microinjected using a 10-μl syringe (Hamilton, Reno, Nevada, US), which was attached to the injection cannula by PE 20 tubing (BTPE-20, Instech Laboratories, Plymouth, Pennsylvania, US). A volume of 0.5 μl per side was injected using a syringe pump (70-3007D, Harvard Apparatus, Holliston, Massachusetts, US) over a 60-second period, and the injection cannula remained untouched for an additional 60 seconds to allow for proper absorption and to minimize pull-up effect along the track of the cannula. Although this rather rapid rate of injection could have increased the probability of diffusion to nearby structures, this is of minor concern because our specific finding has been also shown by others [11,13,41]. The protective cap was secured to the observer animal after the infusion, and then the animal was returned to its home cage.

The shock preexposure for all animals was conducted following the first habituation session. The shock preexposure parameters were identical to those described for experiment 1. All shocks during the shock preexposure were coterminated with a tone stimulus (2.5 kHz, around 70 db, 20 seconds). This tone was then played back to the animals on a later day in a control experiment that is not further reported here.

The testing procedures and parameters for experiment 4 were the same as those described for the familiar condition of experiment 1. Similar to habituation, a white perforated plastic was placed on top of the grid floor of the observer’s chamber.

After completion of the experiment, observer animals were given an overdose of sodium pentobarbital (90 mg/kg, ip). Observers were then intracardially perfused with phosphate-buffered saline (7.4 pH) followed by 4% paraformaldehyde, and brains were removed, cut with a cryostat (50-μm coronal sections), and Nissl stained for verification of cannula position. Four dyads (two from saline group and two from muscimol group) were excluded from data analyses after histological examination suggesting damage of corpus callosum due to injection.

### Behavior scoring

For experiments 1, 2, and 4, the behavior of observers and demonstrators during the interaction test and/or preexposure was manually scored by two experienced researchers (interrater reliability assessed with Pearson’s r correlation coefficient > 0.9) and using the open-source Behavioral Observation Research Interactive Software (BORIS [68]). Freezing in experiment 3 was scored in the same way by another researcher. This third researcher also scored three movies from the original individual familiarity experiment, which revealed very high interrater reliability (r^2^ = 0.99, icc = 0.99) relative to the scorers of the other experiments, ensuring that the data from both experiments can be directly compared. Freezing was defined as lack of movement except for breathing for more than 3 seconds in order to separate brief moments of immobility from long periods of freezing, in line with previous research in rats [60]. Freezing was continuously scored throughout the 12-minute baseline and 12-minute shock periods. To create a continuous time series, freezing moments extracted from the BORIS result files were recoded as 1 and nonfreezing moments as 0 using MATLAB (MathWorks, Natick, Massachusetts, US) on a second-to-second basis. For experiment 4 (i.e., deactivation of the ACC), the researcher that scored the movies was blind to the experimental manipulation (i.e., control or muscimol group). The general motion measurement in experiment 4 was based on the frame-to-frame pixel change in the test area using the activity analysis function in EthoVision XT 11 [69].

### Statistics

#### General linear models

The results of experiments 1, 2, 3, and 4 and the freezing responses of observers and demonstrators were analyzed separately. Freezing time was calculated as the sum of all freezing moments in a certain epoch, and freezing percentage was calculated as the total freezing time divided by the total time of the epoch. Baseline period was defined as the first 710 seconds of the interaction test, and the shock period was defined as the 710 seconds following the first shock (approximately 720 seconds from the start of the test). For comparison between periods and conditions, repeated measures ANOVAs (IBM SPSS, USA) were performed with baseline and shock period as within-subject factors, and the conditions were used as between-subject factors (experiment 1: 0, 1, 3, 5 weeks; experiment 2: same-strain dyads, different-strain dyads; experiment 3: preexposed group, nonpreexposed group; experiment 4: saline group, muscimol group).

#### Bayesian model estimation and comparison

For experiment 1, models were designed using combinations of the following variables: the freezing percent of observers and demonstrators, the number of weeks that observer–demonstrator dyads were housed together (0, 1, 3, 5 weeks), and whether the demonstrators received foot shocks (baseline versus shock period). For experiment 2, models were designed using all possible different combinations of the following variables: the freezing percent of observers and demonstrators, whether observer–demonstrator dyads were from the same (LE-LE, SD-SD) or different strain (LE-SD, SD-LE), whether the demonstrators received foot shocks (baseline versus shock period), the freezing percent of the observers during preexposure, and the strain of the observers and demonstrators (LE versus SD). For experiment 3, the winning models from the individual familiarity experiment of Table 1 was adapted for this purpose by assuming a modulator of preexposure on the connection between demonstrator to observer and observer to demonstrator and allowing for an effect of preexposure directly onto the observer’s freezing.

Note that, in all cases, we only considered the freezing of the other animal during the shock period by multiplying them with the dummy variable Shock_dem_, which had a value of 0 during baseline and 1 when a shock was applied. This was done for two reasons. First, our previous experiments had shown that prior shock experience was necessary for emotional contagion to occur in our paradigm [8], and for the demonstrators, this prior experience was only available after the first shock. Second, inspection of the data (Fig 3) confirmed that the relation between observer and demonstrator freezing that is apparent during the shock period (red pluses) was not apparent during the baseline period (black dots), in which there seemed to be a disconnection between large individual variance in observer freezing (y-axis) and much smaller variance in demonstrator freezing (x-axis). We used relatively flat priors for all parameters with a normal distribution of mean 0 and SD 2. The parameters were initially restricted to real numbers ranging from −1 to 1. For the link between observer and demonstrator freezing, we noticed that estimates sometimes got close to 1. For those parameters, we then relaxed the range to −1.5 to 1.5, and results in the table stem from these less-constrained bounds.

Model fitting and parameter estimation were conducted using Bayesian analysis by estimating the posterior distribution through Bayes’ rule using in-house code in R Stan [70] in R version 3.3.2 (R Core Team, 2016). All models converged (Rhat = 1). To evaluate the predictive accuracy of each model, a leave-one-out cross-validation (Psis_LOO_) was used to estimate the pointwise out-of-sample prediction accuracy (Elpd_loo_) from all the fitted Bayesian models using the log likelihood evaluated at the posterior simulation of the parameter values [71,72]. To select a winning model, models were ranked based on their Elpd_loo_, and differences in model fit were interpreted relative to the SE of the fit. If the model with the highest fit remained within 1 SE of the runner-up models, we inspected whether parameters should be included based on the 90% credibility interval of their posterior distribution. Parameters were included in the winning model if their posterior credibility interval did not include 0, and modulators were included if the 95% credibility intervals of the parameters the modulator splits (e.g., same versus different strain) did not overlap. We repeated the ranking based on Elpd_WAIS_, but this led to identical results.

#### Granger causality analysis

Granger causality is a statistical concept of causality that is based on prediction [73]. If a signal X1 "Granger-causes" (or "G-causes") a signal X2, then past values of X1 should contain information that helps predict X2 above and beyond the information contained in past values of X2 alone. In this study, X1 and X2 were binary time series of freezing of the demonstrator and freezing of the observer (freezing coded as 1 and not freezing coded as 0) on a second-to-second basis. The freezing of the observer at a certain time point (*X2[t]*) can be estimated either by its own history plus a prediction error (reduced model, 1) or also including the history of the freezing of the demonstrator (full model, 2).

$$X2(t)=\sum_{i=1}^{m}A_{X2X2}(i)∙X2(t-i)+\varepsilon (t)$$ (1)

$$X2(t)=\sum_{i=1}^{m}A_{X2X2}^{\prime }(i)∙X2(t-i)+\sum_{i=1}^{m}A_{X1X2}(i)∙X1(t-i)+\varepsilon \prime (t)$$ (2)

In Eq 1 and Eq 2, *t* indicates the different time points (in steps of 1 second), *A* represents the regression coefficients, and *m* refers to the model order, which is the length of the history included. Granger causality from the freezing of the demonstrator to the freezing of the observer (i.e., X1 → X2) is estimated by comparing the full model (Eq 2) to the reduced model (Eq 1). Mathematically, the log likelihood of the two models (i.e., Granger causality value F) is calculated as the natural logarithm of the ratio of the residual covariance matrices of the two models (Eq 3).

$$F_{X1\to X2}=\ln\frac{\left|cov(\varepsilon (t))\right|}{\left|cov(\varepsilon \prime (t))\right|}$$ (3)

This Granger causality magnitude has a natural interpretation in terms of information-theoretic bits-per-unit time [42]. In this study, for example, when Granger causality from the demonstrator to the observer reaches significance, it indicates that the demonstrator's freezing can predict the observer's freezing and that there is information flow from the demonstrator to the observer. Jumping responses of the demonstrator to the foot shocks were also taken into account, and a binary time series of this behavior was included as X3 (jumping coded as 1 and not jumping coded as 0). Given that the demonstrators did not exhibit any jumping during baseline, X3 was only included in the analysis done on the shock period.

The algorithms of the Multivariate Granger Causality (MVGC) Toolbox [42] in MATLAB were used to estimate the magnitude of the Granger causality values. First, the freezing time series of the demonstrators and the observers were smoothed with a Gaussian filter (size = 300 second, sigma = 1.5). The MVGC toolbox confirmed that each time series passed the stationary assumption for Granger causality analysis. Then, the optimal model order (m, the length of history included) was determined by the Akaike Information Criterion (AIC) for the model including all observer–demonstrator dyads. The optimal model order is a balance between maximizing goodness of fit and minimizing the number of coefficients (length of the time series) being estimated. For experiments 1 and 2, the model order of 21 was estimated to be the best fit for the model including all dyads, and thus, it was fixed at 21 for the subsequent dyadwise analysis. The largest model order across all dyads was 22, and running the analysis by fixing the model order to 22 showed similar results. For experiment 4, the estimated best model order was 19, and thus, it was fixed at 19 for the analysis. To test the differences of the Granger causality values across conditions, multivariate ANOVAs were performed using SPSS.

### Simulations

The logic behind the simulations was to explore the hit and false alarm rate of two individuals in a dyad that take decisions to freeze or not to freeze based on an internal danger signal that results from an objective danger signal plus noise. A given time point was considered a hit if the animal froze and danger was present and a false alarm if the animal froze but the danger was absent. Two cases were compared: one in which there is no information exchange between animals (individual case) and one in which there is information flow between animals (social case). In both cases, an animal’s internal danger signal was triggered by witnessing a danger signal d(*t*) that was on for 100 time samples and then off for 100 time samples for five cycles for a total of 1,000 time points:

d(t)=[1..10..01..10..0…]

(a 100 sample–on, 100 sample–off danger cue repeated 5 times).

Both animals experienced noise on top of the signal, with the noise being independent across animals. Noise level was varied systematically by changing σ:

$$n_{i}(t)˜N\left(0,\;\sigma \right)\;\mathrm{with}\;\sigma \epsilon \left[0,10\right]$$

In the no-feedback model, the internal signal of each animal *i* was simply the addition of signal and noise:

$$x_{i}(t)=d\left(t\right)+n_{i}(t)$$

And animals decide to freeze or not to freeze based on whether the signal is above or below threshold c:

$$f_{i}\left(t\right)=1\;if\;x_{i}\left(t\right)>c$$

$$f_{i}\left(t\right)=0\;if\;x_{i}\left(t\right)\leq c$$

In the model with feedback and equal access to the danger signal, we aimed to simulate a situation in which both animals have a similar access to the danger signal but are also sensitive to the freezing of the other animals. We thus calculated the internal signal iteratively by additionally considering whether the other animal froze on the preceding time point, with both animals experiencing equal noise levels. The degree to which the internal signal depends on the freezing of the other is systematically varied using bϵ[0,2]. Given that both the danger signal and the freezing of the other animal take on values of 0 and 1, b = 1 means that the animal pays equal attention to sensory and social sources of information:

$$x_{1}\left(t\right)=d\left(t\right)+n_{1}\left(t\right)+bȥ\cdot (f_{2}\left[t-1\right]-0.5)$$

$$x_{2}\left(t\right)=d\left(t\right)+n_{2}\left(t\right)+b\cdot (f_{1}\left[t-1\right]-0.5)$$

$$f_{i}\left(t\right)=1\;if\;x_{i}\left(t\right)>c$$

$$f_{i}\left(t\right)=0\;if\;x_{i}\left(t\right)<c$$

Finally, in models with feedback but unequal access to the danger signal, we aimed to simulate conditions in which one animal has more access to the danger signal than the other by adding r times more noise to animal 1 than 2. In that case, the degree to which the two animals consider the freezing from the other is scaled based on experienced noise, with animals experiencing more noise paying more attention to the freezing in the other. This decision was informed by our finding that demonstrators are less influenced by observers than vice versa and by the finding that humans integrate the influence of others in similar ways [74].

$$x_{1}\left(t\right)=d\left(t\right)+r*n_{1}\left(t\right)+b\cdot (f_{2}\left[t-1\right)-0.5)$$

$$x_{2}\left(t\right)=d\left(t\right)+n_{2}\left(t\right)+b/r\cdot (f_{1}\left[t-1\right]-0.5)$$

$$f_{i}\left(t\right)=1\;if\;x_{i}\left(t\right)>c$$

$$f_{i}\left(t\right)=0\;if\;x_{i}\left(t\right)<c$$

Performance was measured based on signal detection theory as the area under the ROC curve. Specifically, c is varied systematically from −5σ to +5σ, and the hit and false alarm rate is calculated in each case, with a hit being a freezing decision when the danger signal was 1 and a false alarm when it was 0. These rates are then plotted on an ROC curve, with false alarms on x and hits on y coordinates. Random decisions lead to AUC of 0.5, and perfect decisions lead to AUC = 1. The gain in performance between the individual and social condition was calculated as (AUC_social_ − AUC_individual_)/(AUC_individual_ − 0.5) to express how much further from chance the performance has become.

To explore more systematically the influence of noise level (σ), coupling (b), and noise ratio (r), for each combination of parameters, we calculated performance gains 20 times (using new random numbers for the noise) and display the median of these 20 random noise sets.

## Abbreviations

ACC: anterior cingulate cortex
AUC: area under the curve
cg: cingulate gyrus
dem: demonstrator
Elpd_loo_: expected log pointwise predictive density according to the leave-one-out approximation
ISI: intershock interval
LV: lateral ventricle
obs: observer
ROC: receiver operating characteristic
SE: standard error
SEM: standard error of the mean
